# Supplementary material for: An Optimal Control Framework for the Automated Design of Personalized Cancer Treatments
Source: Front Bioeng Biotechnol. 2020 May 28;8:523. doi: 10.3389/fbioe.2020.00523 (PMC7270334; doi:10.3389/fbioe.2020.00523)
Supplement: Supplementary file 1 [file Data_Sheet_1.pdf]

Supplementary Materials: An optimal control framework for the automated design of personalized cancer treatments

1 OPTIMAL SCHEDULE

Analogously, we can optimize also the schedule of administration $(t_0^*, t_1^*, ..., t_n^*)$ with a new pulse $u_2(t)$. So we can introduce a new pulse $T(t) = u_2(t)$ and using a similar integral structure as before:

$$
\begin{aligned}
&t_0^* = t_0 \\
&t_1^* = t_0^* + \int_{t_0}^{t_1} dt^{'}\tilde{T}(t^{'}) \\
&\vdots \\
&t_j^* = t_{j-1}^* + \int_{t_{j-1}}^{t_j} dt^{'}\tilde{T}(t^{'}) \\
&\vdots \\
&t_n^* = t_{n-1}^* + \int_{t_{n-1}}^{t_n} dt^{'}\tilde{T}(t^{'}) \\
&t_f^* = t_n^* + \int_{t_n}^{t_f} dt^{'}\tilde{T}(t^{'}) = t_f
\end{aligned}
\tag{S1}
$$

where $\tilde{T}(t)$ is the normalized function with respect to the difference between the initial time t_0 and t_f, and is defined as follows:

$$
\tilde{T}(t) = \frac{T(t)}{\int_{t_0}^{t_f} dt' T(t')} \left(t_0 - t_f\right) . \tag{S2}
$$

So in this case the set of controls used are the set dosages D_i^* and the set t_i^* of times when drug is administered in Eq. (12) of the main text: $\chi_{gi}(t) = f D_i(t_i) e^{-ka(t-t_i)}$, as in the case presented in the main text it is necessary to fix n *a priori*.

Note that we here do not set any constrain about the minimum and the maximum time gap between two administrations. However, as drug holiday periods are supposed to be relevant for the health of the patient and for the success of a therapy, this aspect deserves further investigations (Castiglione and Piccoli (2007))

2 FIGURES

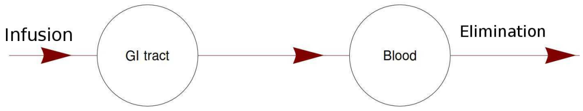

Figure S1: Diagram of compartmental PK for oral administration of Imatinib.

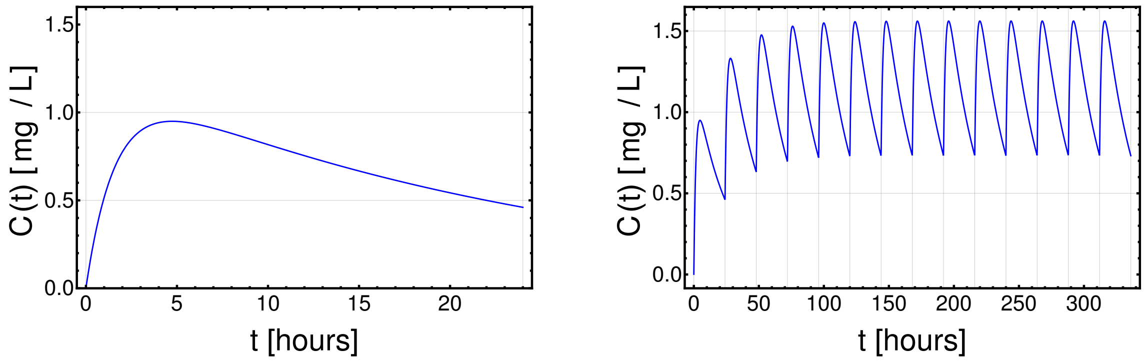

Figure S2: On the left is the simulation of the concentration in the blood $C(t)$ of a single oral administration of $400mg$ of Imanitib. On the right there is a simulation of a multi-dose administration of $400mg$ of Imanitib, the drug is taken every 24 hours. Parameters of these simulation are presented in table S1

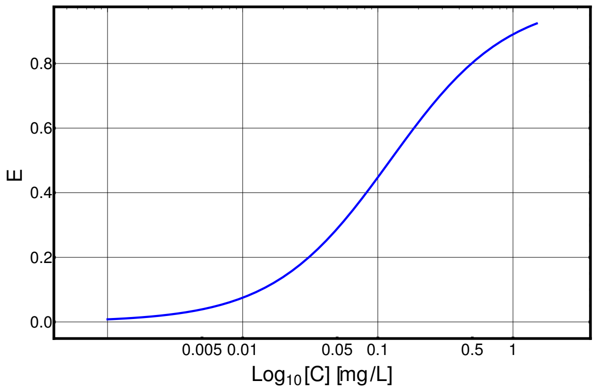

Figure S3: Curve of efficacy given by Eq. (9), we impose $n = 1$, $EC_{50} = 0.123 \quad [mg/L]$ and $E_{max} = 1$.

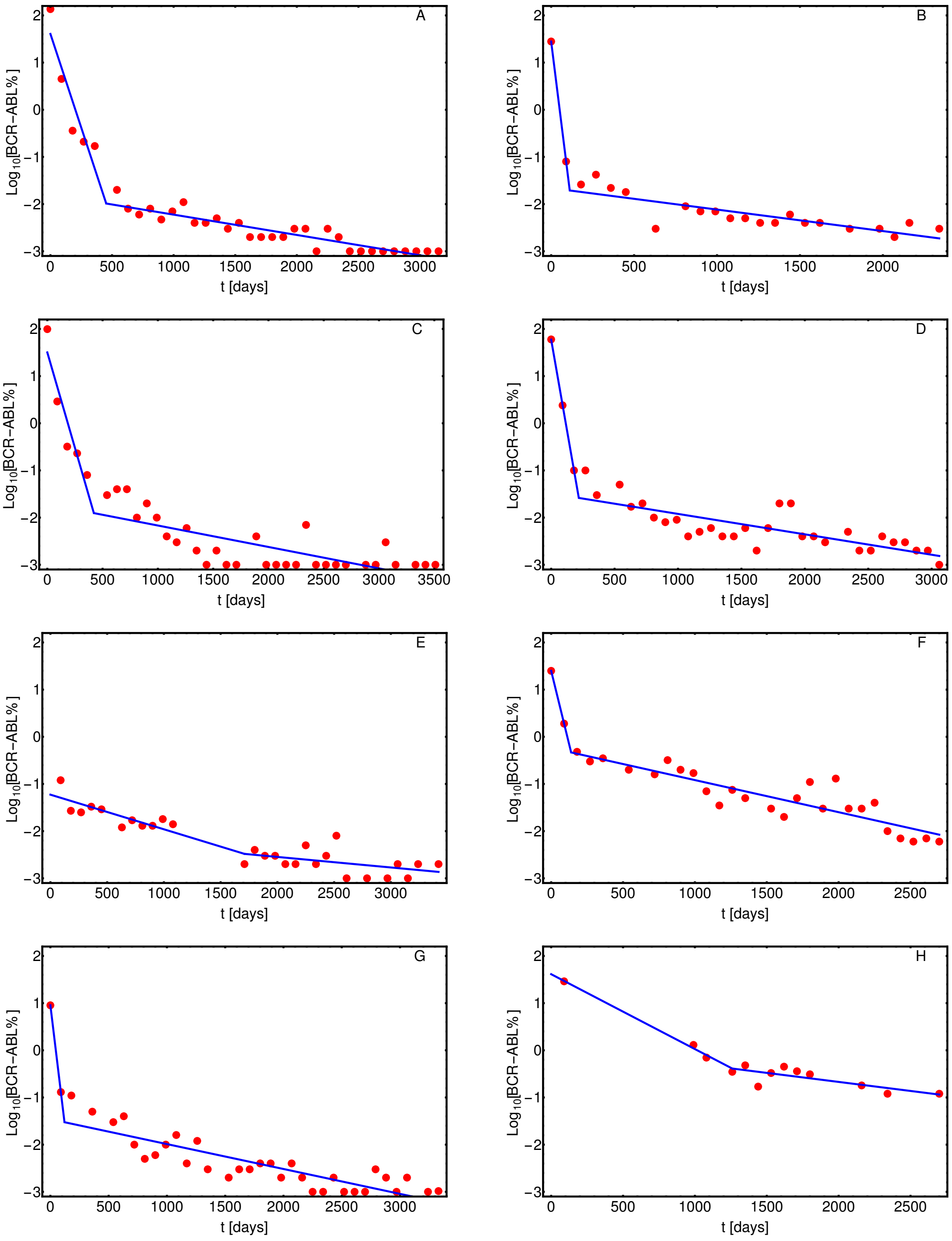

Figure S4: Data analysis. The red dots are the experimental points and they represents the expression of BCR-ABL. Blue lines are the best fits. Results of the data analysis are summarized in table S3.

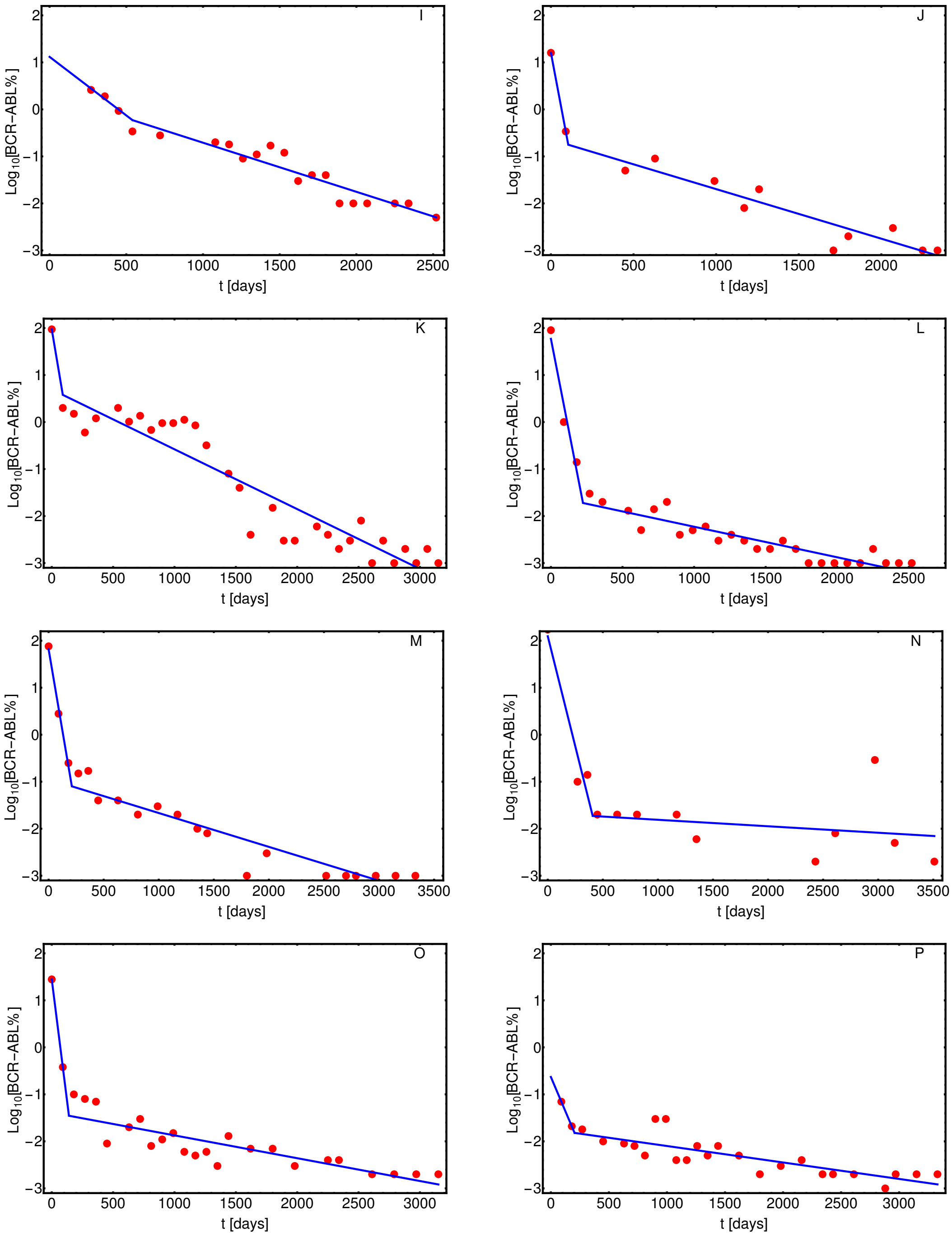

Figure S5: Data analysis. The red dots are the experimental points and they represents the expression of BCR-ABL. Blue lines are the best fits. Results of the data analysis are summarized in table S3

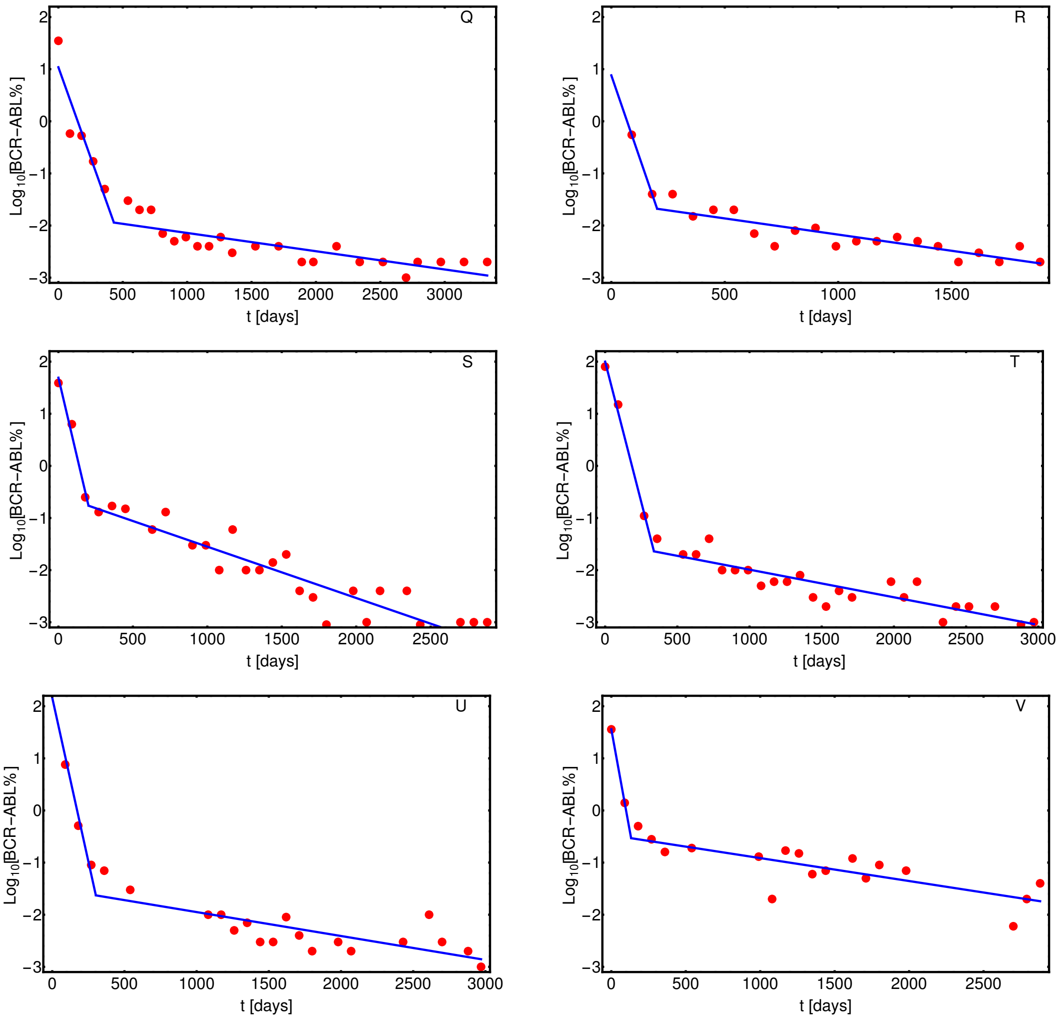

Figure S6: Data analysis. The red dots are the experimental points and they represents the expression of BCR-ABL. Blue lines are the best fits. Results of the data analysis are summarized in table S3

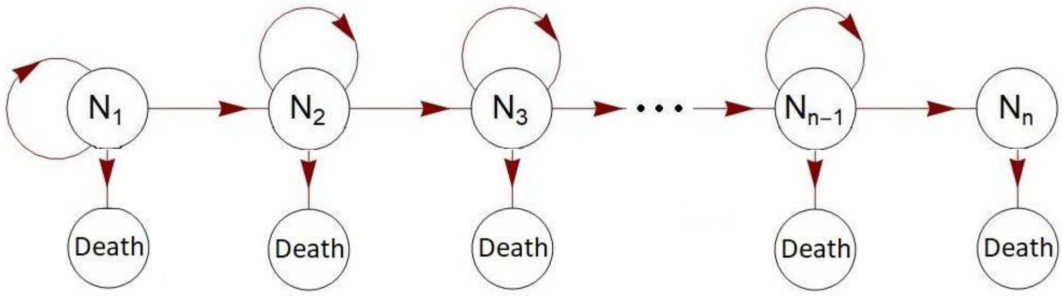

Figure S7: Diagram of compartmental model for an unregulated leukemic cells lineage.

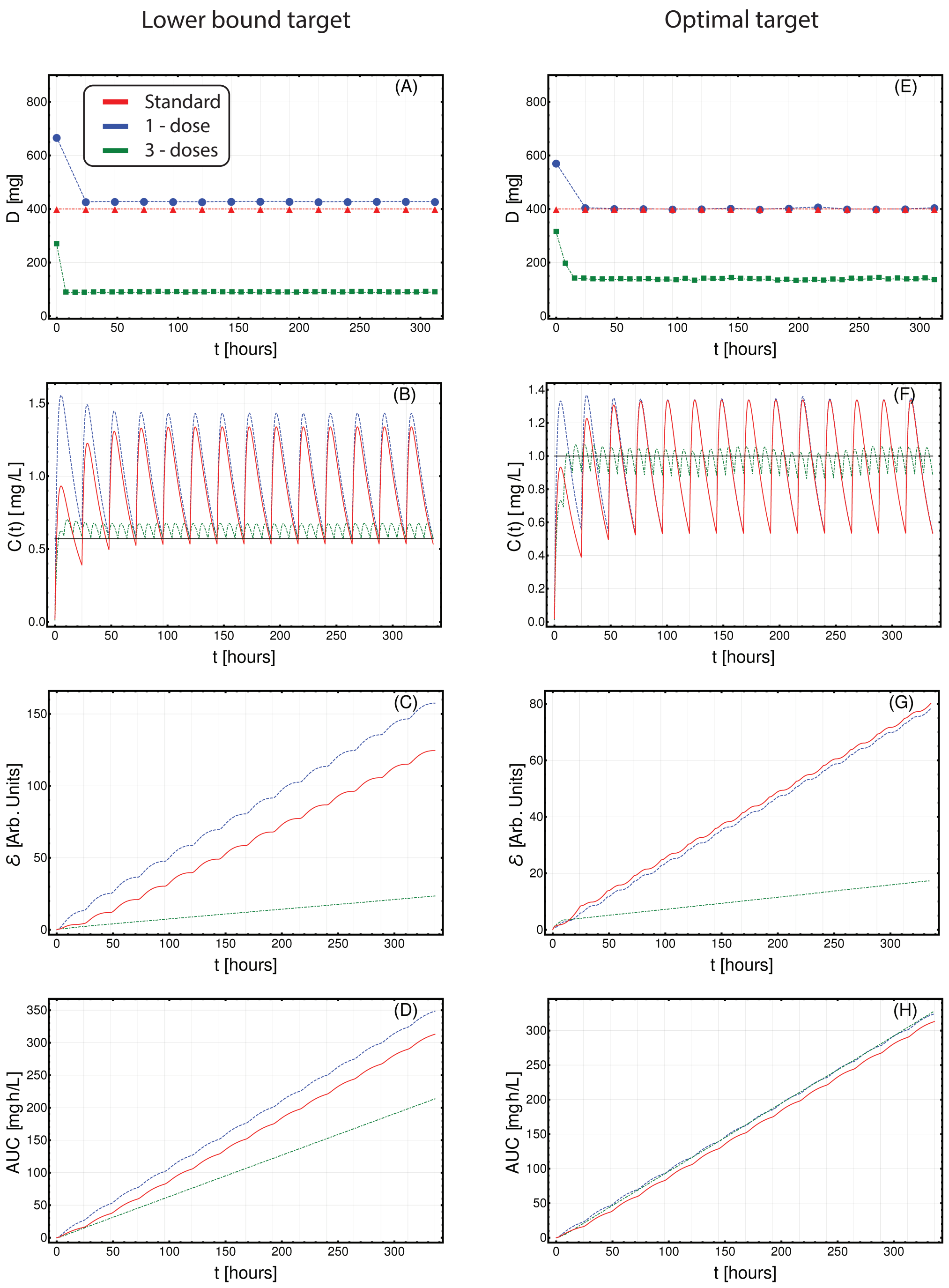

Figure S8: Optimized Imatinib administration returned by CT4TD for patient 0001 00002 RH from (Michor et al. (2005)), in the cases of: 1-dose/day (blue) and 3-doses/day (green), with respect to: *lower-bound* target concentration $C_{targ} = 0.57$ $[mg/L]$ (left panels: A–D) and *optimal* target concentration $C_{targ} = 1$ $[mg/L]$ (right panels: E–H). Standard administration – i.e., 400 mg Imatinib/day – is shown with a red dashed line. In this case, the optimization of PK/PD models is obtained on patient-specific PK parameters, without considering the PD models. **(A,D)** Imatinib scheduled dosage in mg (y axis), displayed on 14 days (x axis). **(B-E)** Imatinib concentration in blood in $[mg/L]$ (y axis). **(C-F)** Variation of the cumulative distance between the observed concentration and the target in time.**(D-H)** The AUC in $[mg \cdot h/L]$

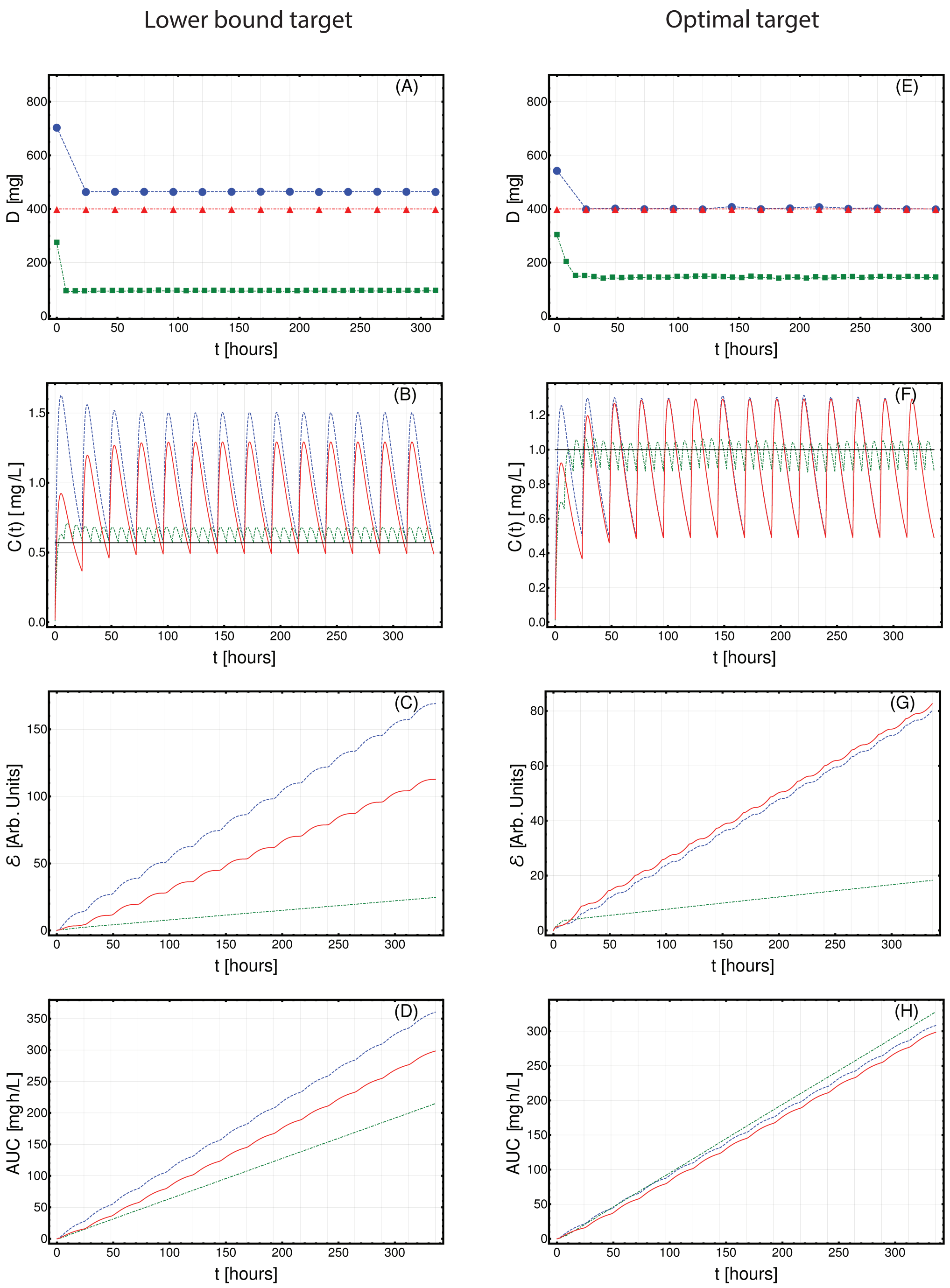

Figure S9: Optimized Imatinib administration returned by CT4TD for patient 0001 00006 GMC from (Michor et al. (2005)), in the cases of: 1-dose/day (blue) and 3-doses/day (green), with respect to: *lower-bound* target concentration $C_{targ} = 0.57\ [mg/L]$ (left panels: A–D) and *optimal* target concentration $C_{targ} = 1\ [mg/L]$ (right panels: E–H). Standard administration – i.e., 400 mg Imatinib/day – is shown with a red dashed line. In this case, the optimization of PK/PD models is obtained on patient-specific PK parameters, without considering the PD models. **(A,D)** Imatinib scheduled dosage in mg (y axis), displayed on 14 days (x axis). **(B-E)** Imatinib concentration in blood in $[mg/L]$ (y axis). **(C-F)** Variation of the cumulative distance between the observed concentration and the target in time.**(D-H)** The AUC in $[mg \cdot h/L]$

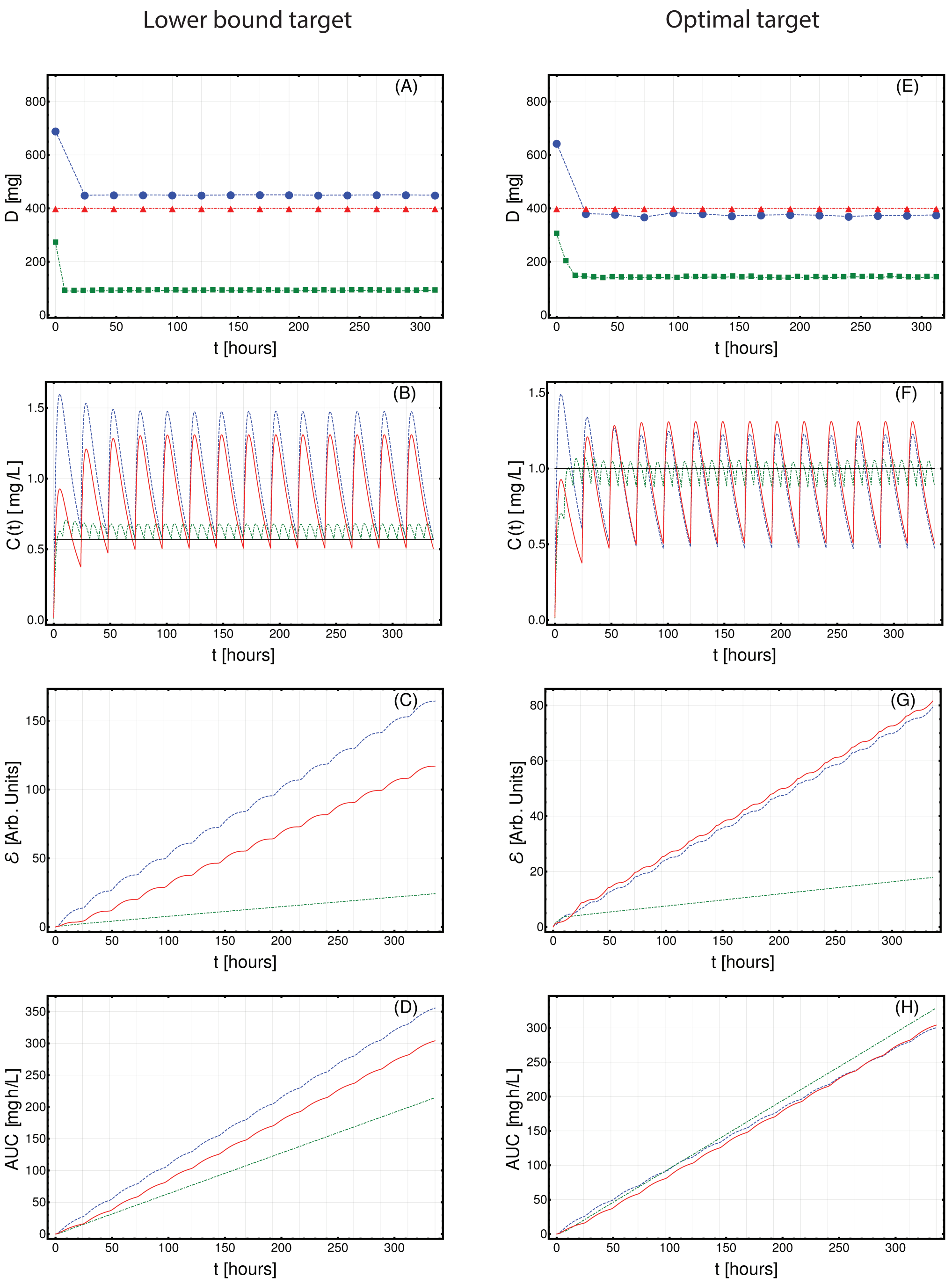

Figure S10: Optimized Imatinib administration returned by CT4TD for patient 0001 00009 MJG from (Michor et al. (2005)), in the cases of: 1-dose/day (blue) and 3-doses/day (green), with respect to: *lower-bound* target concentration $C_{targ} = 0.57$ $[mg/L]$ (left panels: A–D) and *optimal* target concentration $C_{targ} = 1$ $[mg/L]$ (right panels: E–H). Standard administration – i.e., 400 mg Imatinib/day – is shown with a red dashed line. In this case, the optimization of PK/PD models is obtained on patient-specific PK parameters, without considering the PD models. **(A,D)** Imatinib scheduled dosage in mg (y axis), displayed on 14 days (x axis). **(B-E)** Imatinib concentration in blood in $[mg/L]$ (y axis). **(C-F)** Variation of the cumulative distance between the observed concentration and the target in time.**(D-H)** The AUC in $[mg \cdot h/L]$

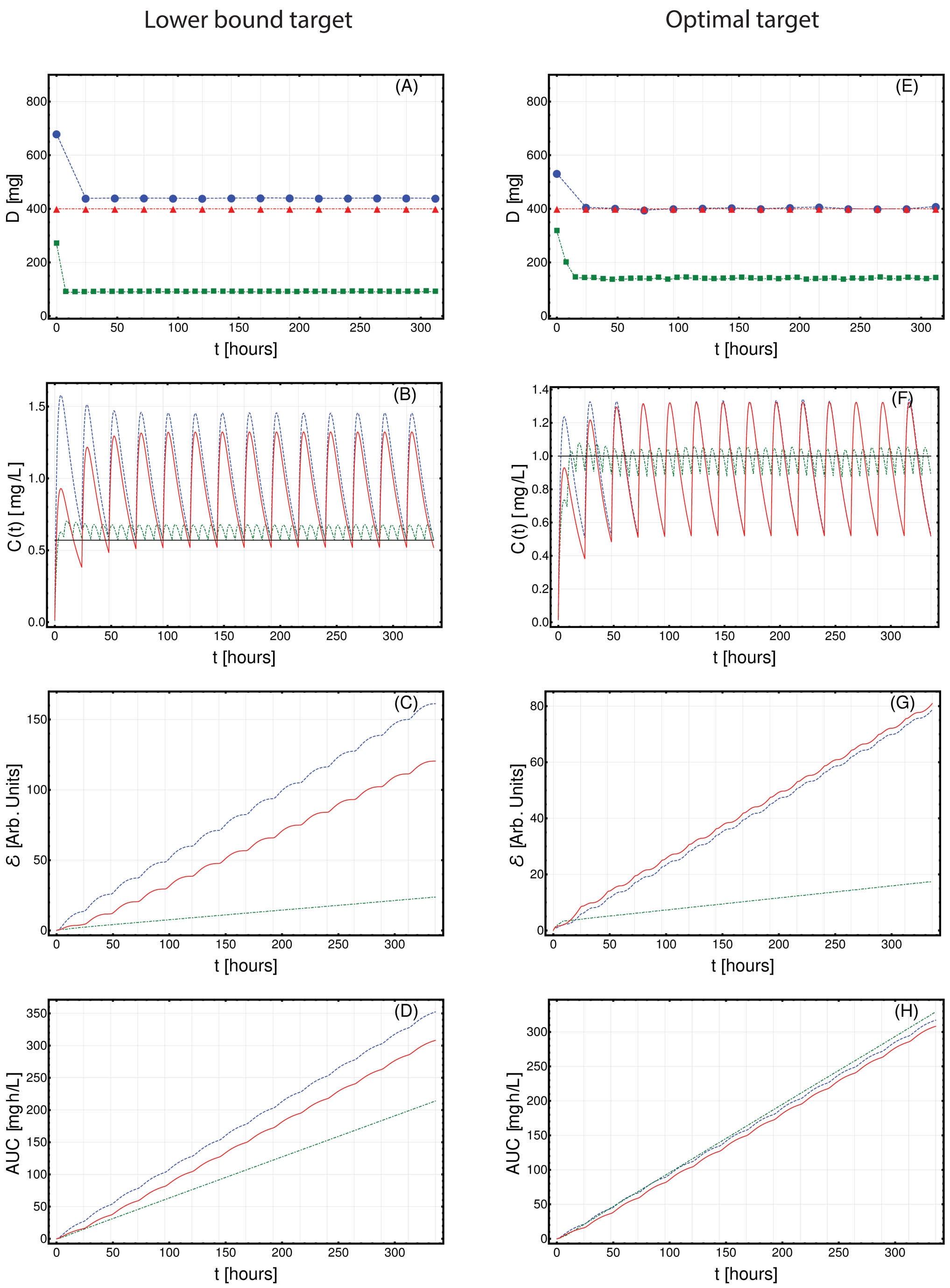

Figure S11: Optimized Imatinib administration returned by CT4TD for patient 0002 00003 SMV from (Michor et al. (2005)), in the cases of: 1-dose/day (blue) and 3-doses/day (green), with respect to: *lower-bound* target concentration $C_{targ} = 0.57\ [mg/L]$ (left panels: A–D) and *optimal* target concentration $C_{targ} = 1\ [mg/L]$ (right panels: E–H). Standard administration – i.e., 400 mg Imatinib/day – is shown with a red dashed line. In this case, the optimization of PK/PD models is obtained on patient-specific PK parameters, without considering the PD models. **(A,D)** Imatinib scheduled dosage in mg (y axis), displayed on 14 days (x axis). **(B-E)** Imatinib concentration in blood in $[mg/L]$ (y axis). **(C-F)** Variation of the cumulative distance between the observed concentration and the target in time.**(D-H)** The AUC in $[mg \cdot h/L]$

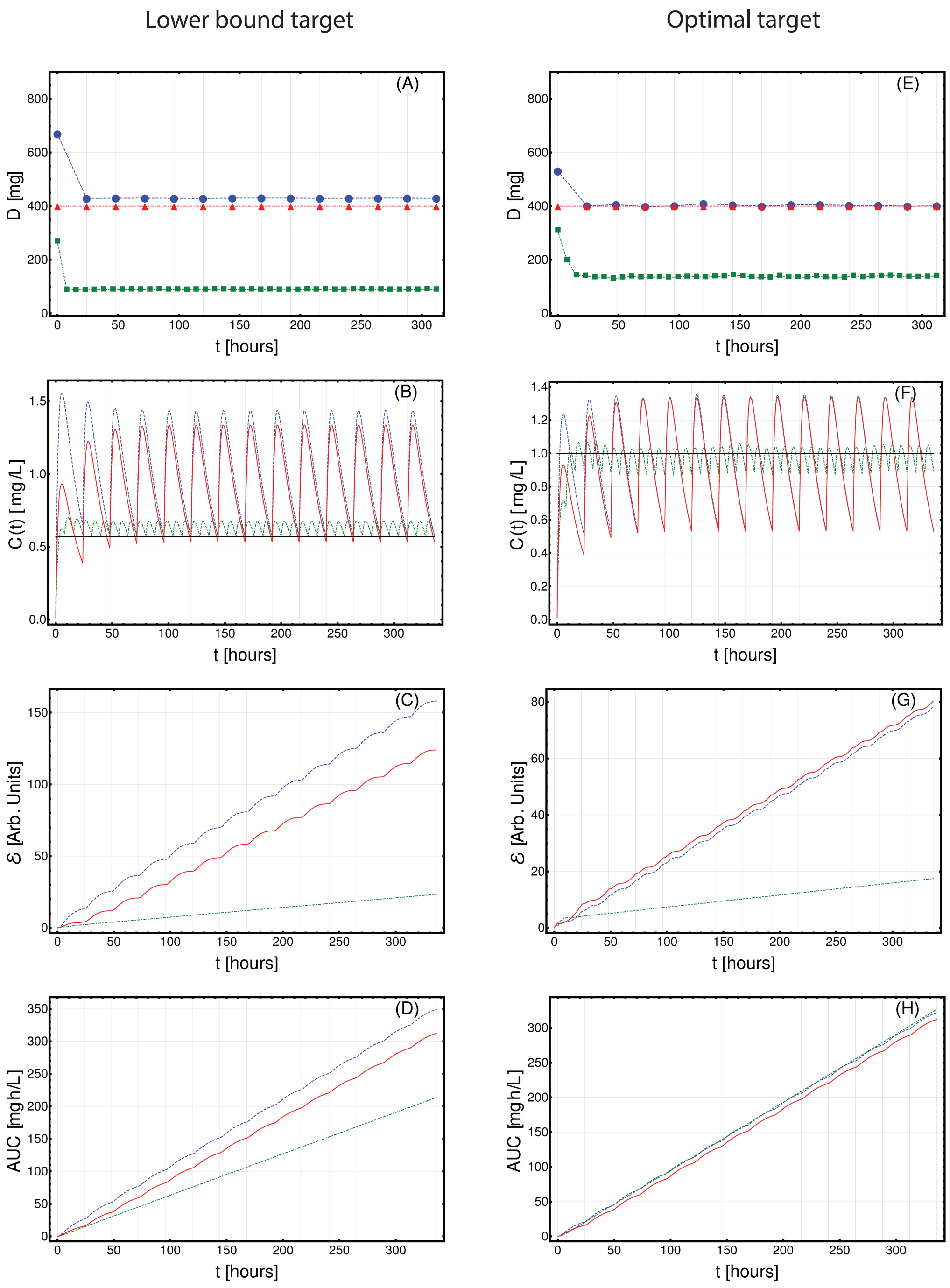

Figure S12: Optimized Imatinib administration returned by CT4TD for patient 0002 00003 PMG from (Michor et al. (2005)), in the cases of: 1-dose/day (blue) and 3-doses/day (green), with respect to: *lower-bound* target concentration $C_{targ} = 0.57$ $[mg/L]$ (left panels: A–D) and *optimal* target concentration $C_{targ} = 1$ $[mg/L]$ (right panels: E–H). Standard administration – i.e., 400 mg Imatinib/day – is shown with a red dashed line. In this case, the optimization of PK/PD models is obtained on patient-specific PK parameters, without considering the PD models. **(A,D)** Imatinib scheduled dosage in mg (y axis), displayed on 14 days (x axis). **(B-E)** Imatinib concentration in blood in $[mg/L]$ (y axis). **(C-F)** Variation of the cumulative distance between the observed concentration and the target in time.**(D-H)** The AUC in $[mg \cdot h/L]$

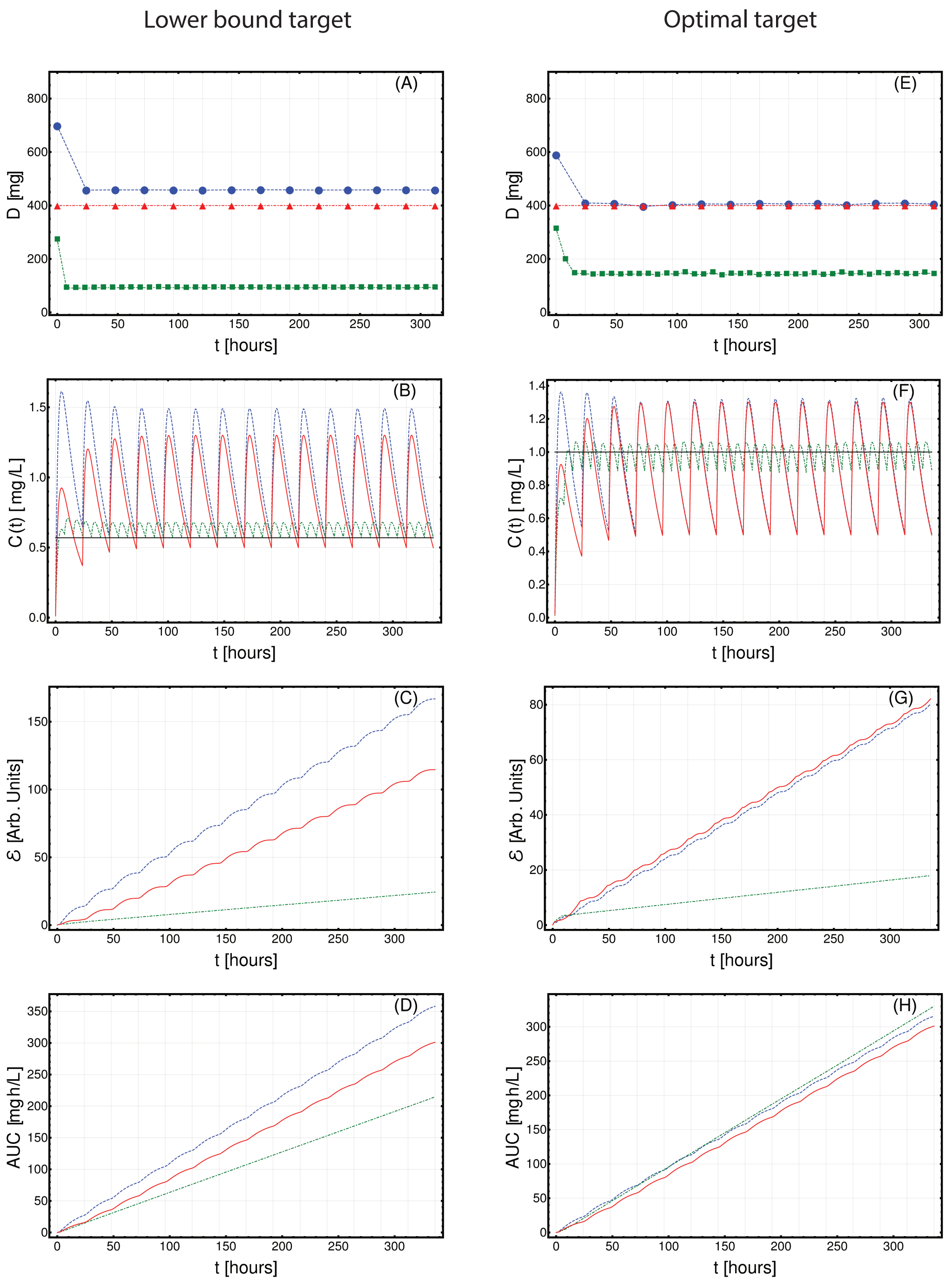

Figure S13: Optimized Imatinib administration returned by CT4TD for patient 0002 00008 SAT from (Michor et al. (2005)), in the cases of: 1-dose/day (blue) and 3-doses/day (green), with respect to: *lower-bound* target concentration $C_{targ} = 0.57\ [mg/L]$ (left panels: A–D) and *optimal* target concentration $C_{targ} = 1\ [mg/L]$ (right panels: E–H). Standard administration – i.e., 400 mg Imatinib/day – is shown with a red dashed line. In this case, the optimization of PK/PD models is obtained on patient-specific PK parameters, without considering the PD models. **(A,D)** Imatinib scheduled dosage in mg (y axis), displayed on 14 days (x axis). **(B-E)** Imatinib concentration in blood in $[mg/L]$ (y axis). **(C-F)** Variation of the cumulative distance between the observed concentration and the target in time.AF **(D-H)** The AUC in $[mg \cdot h/L]$

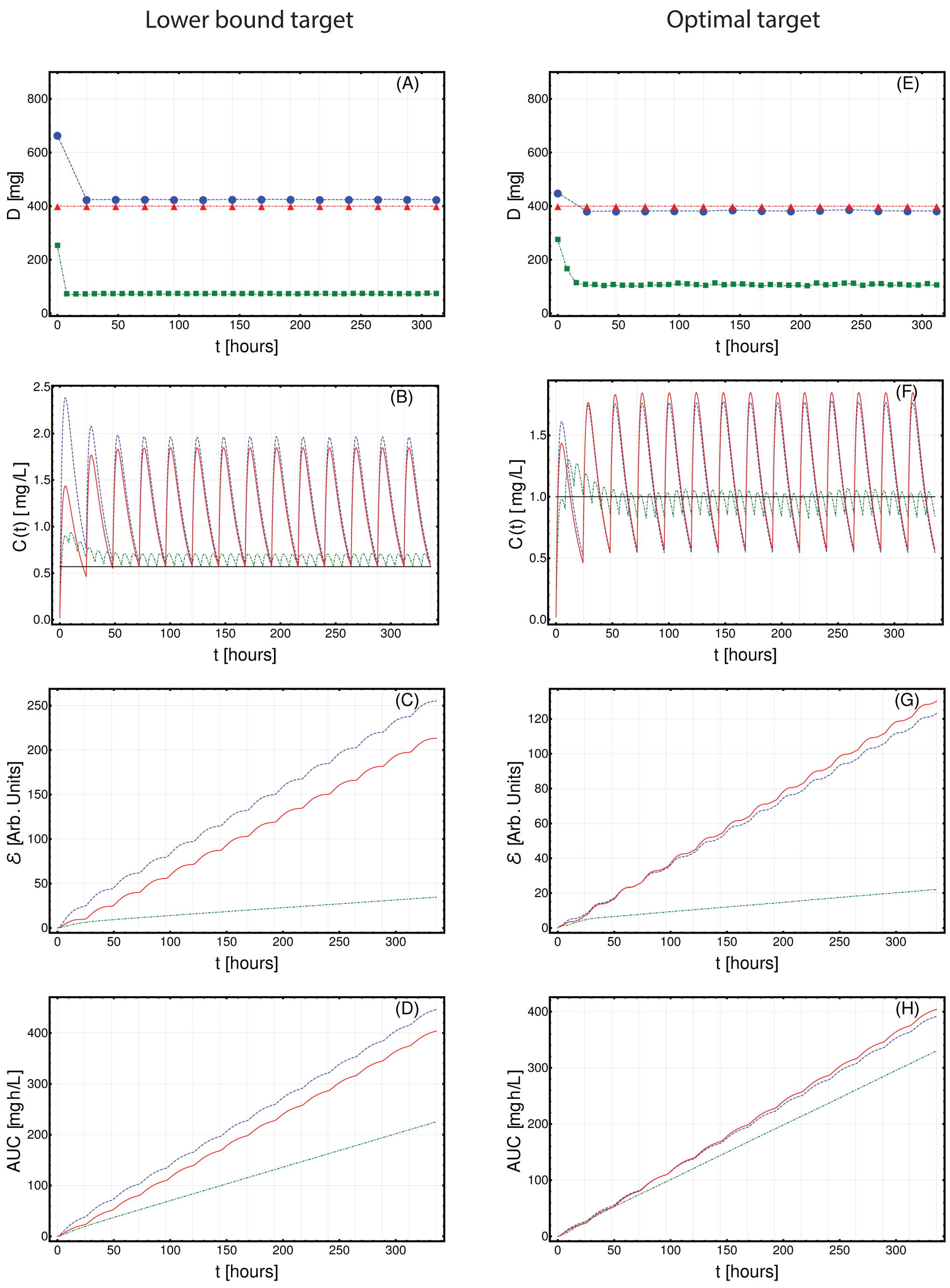

Figure S14: Optimized Imatinib administration returned by CT4TD for patient 0003 00002 CL from (Michor et al. (2005)), in the cases of: 1-dose/day (blue) and 3-doses/day (green), with respect to: *lower-bound* target concentration $C_{targ} = 0.57\ [mg/L]$ (left panels: A–D) and *optimal* target concentration $C_{targ} = 1\ [mg/L]$ (right panels: E–H). Standard administration – i.e., 400 mg Imatinib/day – is shown with a red dashed line. In this case, the optimization of PK/PD models is obtained on patient-specific PK parameters, without considering the PD models. **(A,D)** Imatinib scheduled dosage in mg (y axis), displayed on 14 days (x axis). **(B-E)** Imatinib concentration in blood in $[mg/L]$ (y axis). **(C-F)** Variation of the cumulative distance between the observed concentration and the target in time.**(D-H)** The AUC in $[mg \cdot h/L]$

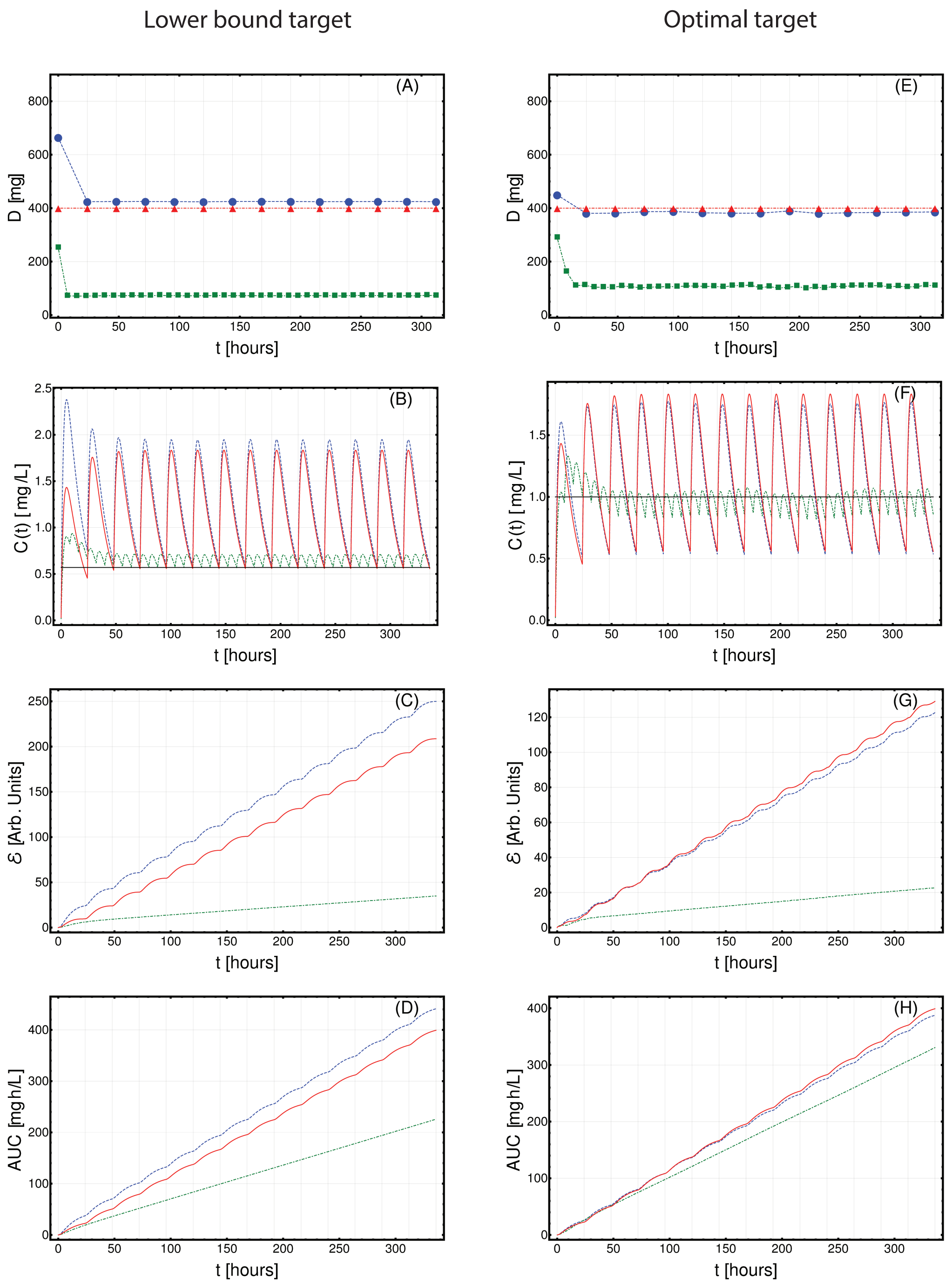

Figure S15: Optimized Imatinib administration returned by CT4TD for patient 0004 00003 CAR from (Michor et al. (2005)), in the cases of: 1-dose/day (blue) and 3-doses/day (green), with respect to: *lower-bound* target concentration $C_{targ} = 0.57\ [mg/L]$ (left panels: A–D) and *optimal* target concentration $C_{targ} = 1\ [mg/L]$ (right panels: E–H). Standard administration – i.e., 400 mg Imatinib/day – is shown with a red dashed line. In this case, the optimization of PK/PD models is obtained on patient-specific PK parameters, without considering the PD models. **(A,D)** Imatinib scheduled dosage in mg (y axis), displayed on 14 days (x axis). **(B-E)** Imatinib concentration in blood in $[mg/L]$ (y axis). **(C-F)** Variation of the cumulative distance between the observed concentration and the target in time.**(D-H)** The AUC in $[mg \cdot h/L]$

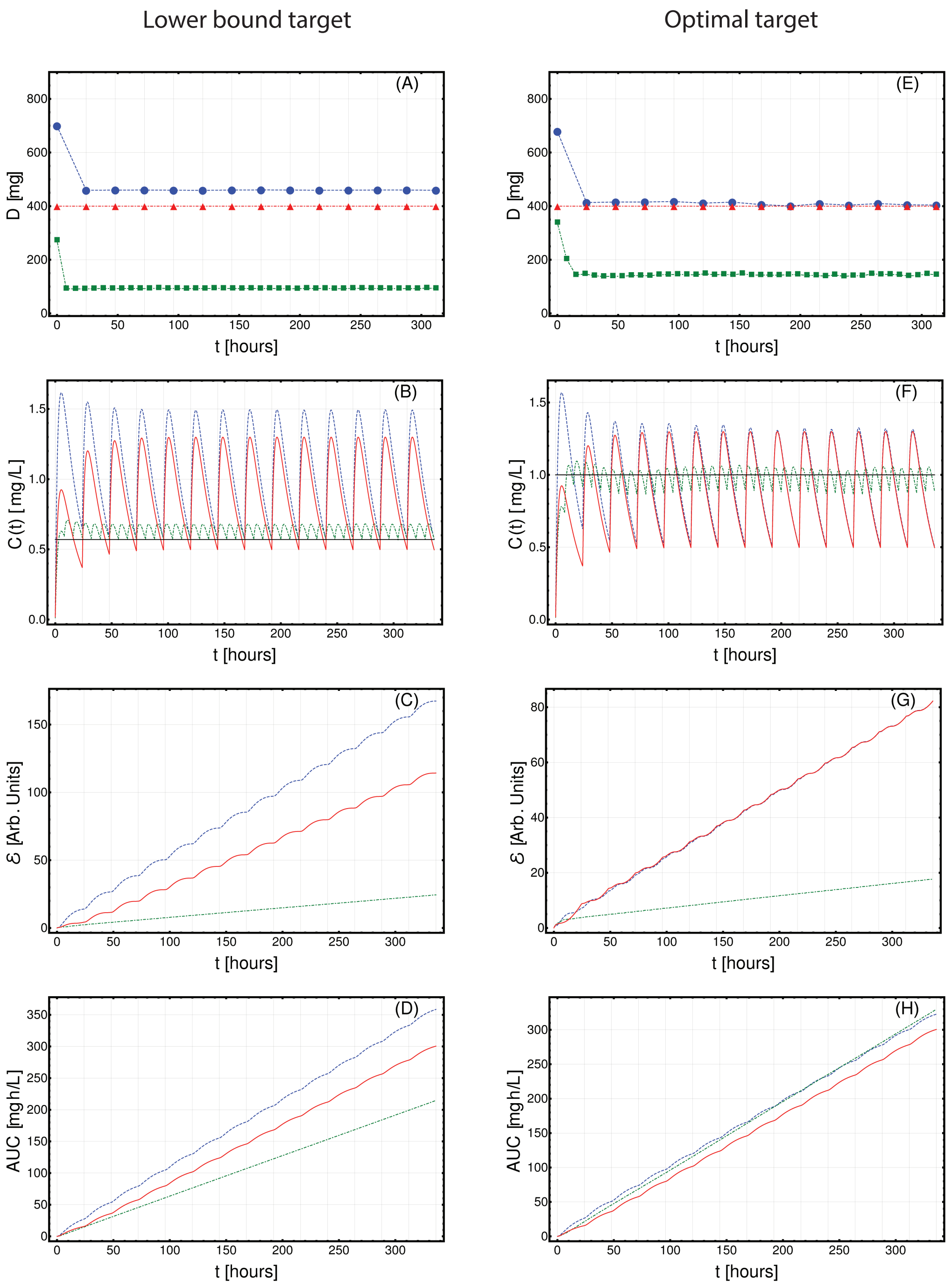

Figure S16: Optimized Imatinib administration returned by CT4TD for patient 0004 00006 GM from (Michor et al. (2005)), in the cases of: 1-dose/day (blue) and 3-doses/day (green), with respect to: *lower-bound* target concentration $C_{targ} = 0.57\ [mg/L]$ (left panels: A–D) and *optimal* target concentration $C_{targ} = 1\ [mg/L]$ (right panels: E–H). Standard administration – i.e., 400 mg Imatinib/day – is shown with a red dashed line. In this case, the optimization of PK/PD models is obtained on patient-specific PK parameters, without considering the PD models. **(A,D)** Imatinib scheduled dosage in mg (y axis), displayed on 14 days (x axis). **(B-E)** Imatinib concentration in blood in $[mg/L]$ (y axis). **(C-F)** Variation of the cumulative distance between the observed concentration and the target in time.**(D-H)** The AUC in $[mg \cdot h/L]$

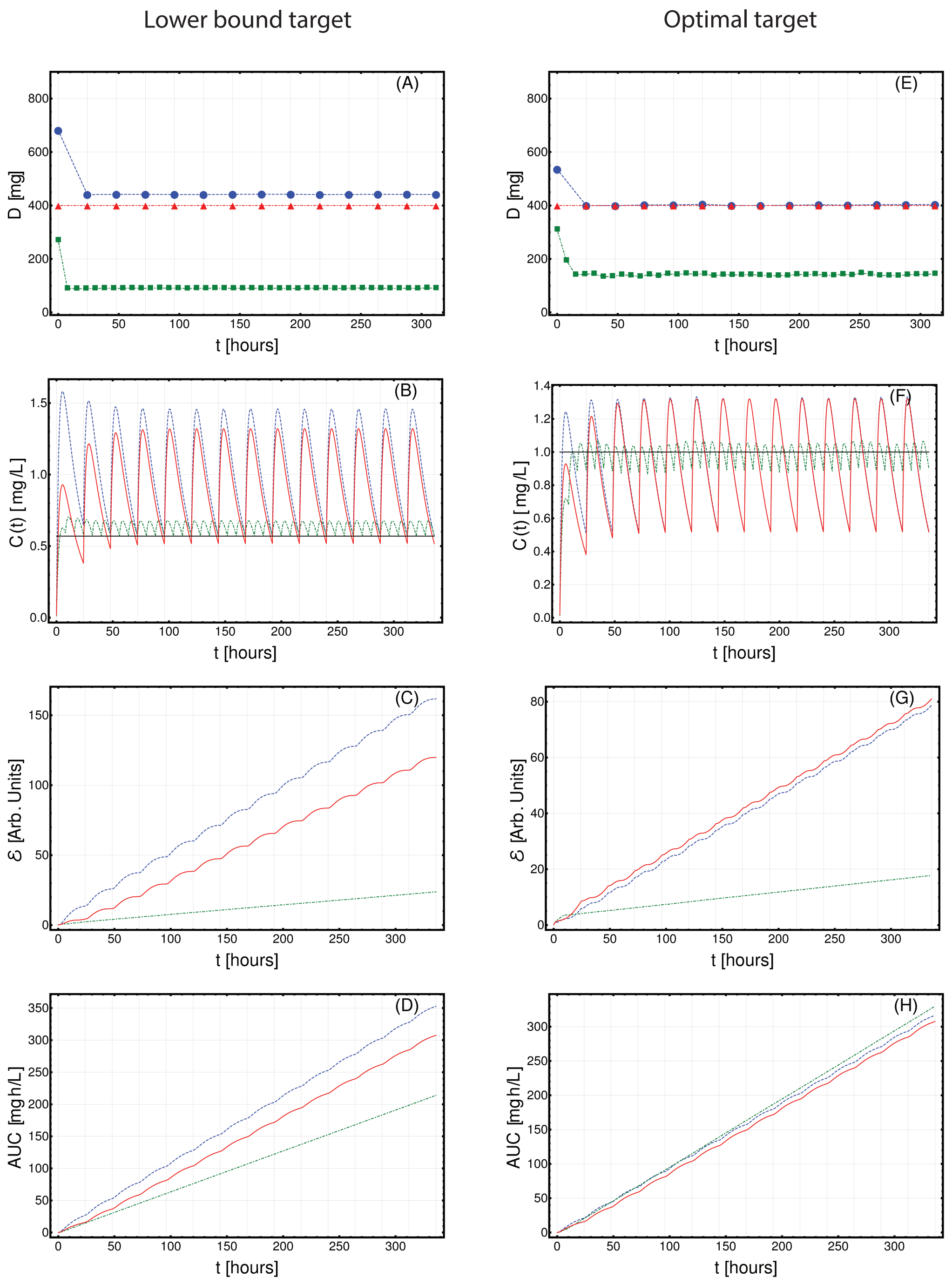

Figure S17: Optimized Imatinib administration returned by CT4TD for patient 0006 00006 ML from (Michor et al. (2005)), in the cases of: 1-dose/day (blue) and 3-doses/day (green), with respect to: *lower-bound* target concentration $C_{targ} = 0.57\ [mg/L]$ (left panels: A–D) and *optimal* target concentration $C_{targ} = 1\ [mg/L]$ (right panels: E–H). Standard administration – i.e., 400 mg Imatinib/day – is shown with a red dashed line. In this case, the optimization of PK/PD models is obtained on patient-specific PK parameters, without considering the PD models. **(A,D)** Imatinib scheduled dosage in mg (y axis), displayed on 14 days (x axis). **(B-E)** Imatinib concentration in blood in $[mg/L]$ (y axis). **(C-F)** Variation of the cumulative distance between the observed concentration and the target in time.**(D-H)** The AUC in $[mg \cdot h/L]$

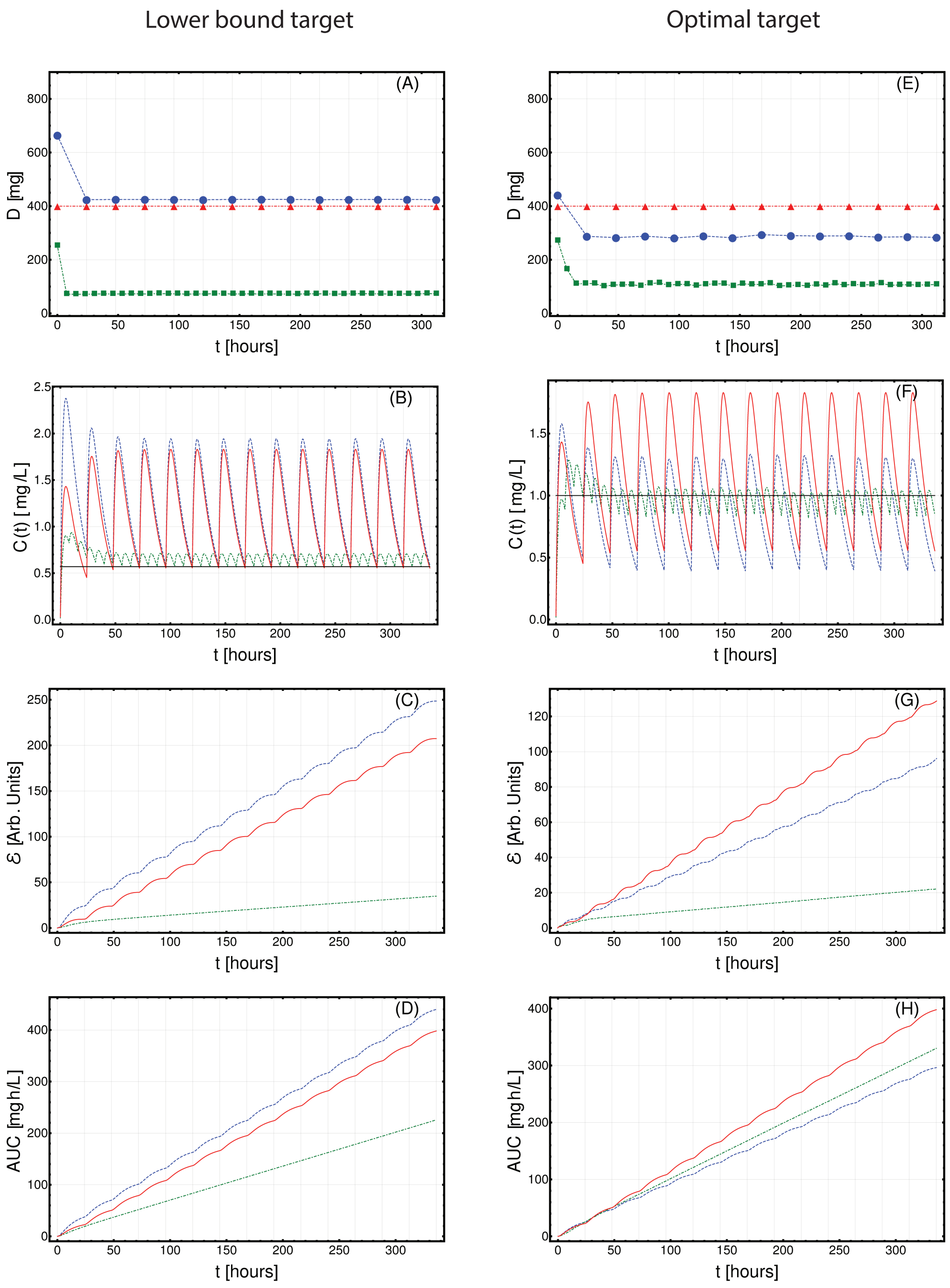

Figure S18: Optimized Imatinib administration returned by CT4TD for patient 0006 00007 RJW from (Michor et al. (2005)), in the cases of: 1-dose/day (blue) and 3-doses/day (green), with respect to: *lower-bound* target concentration $C_{targ} = 0.57\ [mg/L]$ (left panels: A–D) and *optimal* target concentration $C_{targ} = 1\ [mg/L]$ (right panels: E–H). Standard administration – i.e., 400 mg Imatinib/day – is shown with a red dashed line. In this case, the optimization of PK/PD models is obtained on patient-specific PK parameters, without considering the PD models. **(A,D)** Imatinib scheduled dosage in mg (y axis), displayed on 14 days (x axis). **(B-E)** Imatinib concentration in blood in $[mg/L]$ (y axis). **(C-F)** Variation of the cumulative distance between the observed concentration and the target in time.**(D-H)** The AUC in $[mg \cdot h/L]$

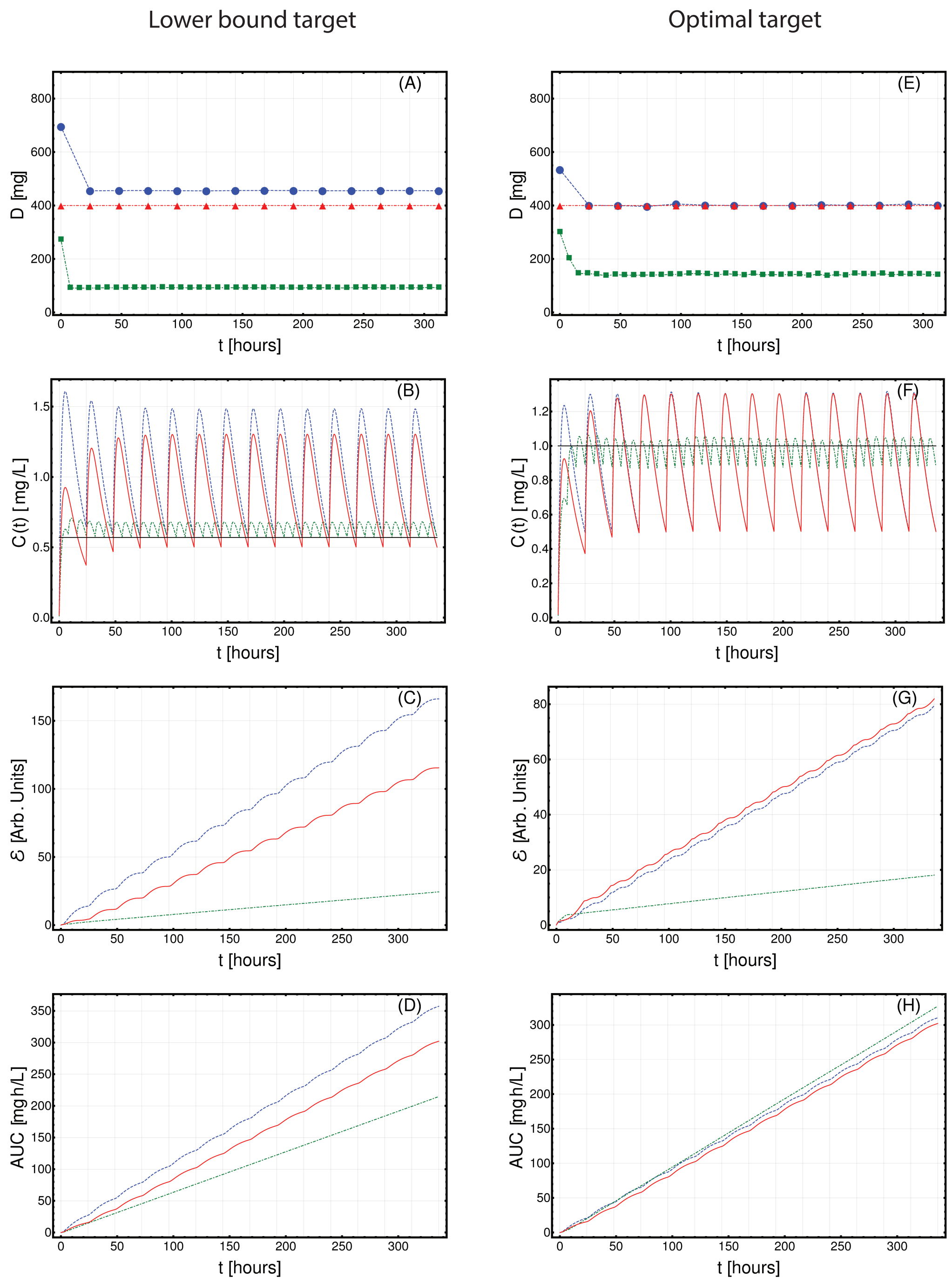

Figure S19: Optimized Imatinib administration returned by CT4TD for patient 0007 00001 AJV from (Michor et al. (2005)), in the cases of: 1-dose/day (blue) and 3-doses/day (green), with respect to: *lower-bound* target concentration $C_{targ} = 0.57\ [mg/L]$ (left panels: A–D) and *optimal* target concentration $C_{targ} = 1\ [mg/L]$ (right panels: E–H). Standard administration – i.e., 400 mg Imatinib/day – is shown with a red dashed line. In this case, the optimization of PK/PD models is obtained on patient-specific PK parameters, without considering the PD models. **(A,D)** Imatinib scheduled dosage in mg (y axis), displayed on 14 days (x axis). **(B-E)** Imatinib concentration in blood in $[mg/L]$ (y axis). **(C-F)** Variation of the cumulative distance between the observed concentration and the target in time.**(D-H)** The AUC in $[mg \cdot h/L]$

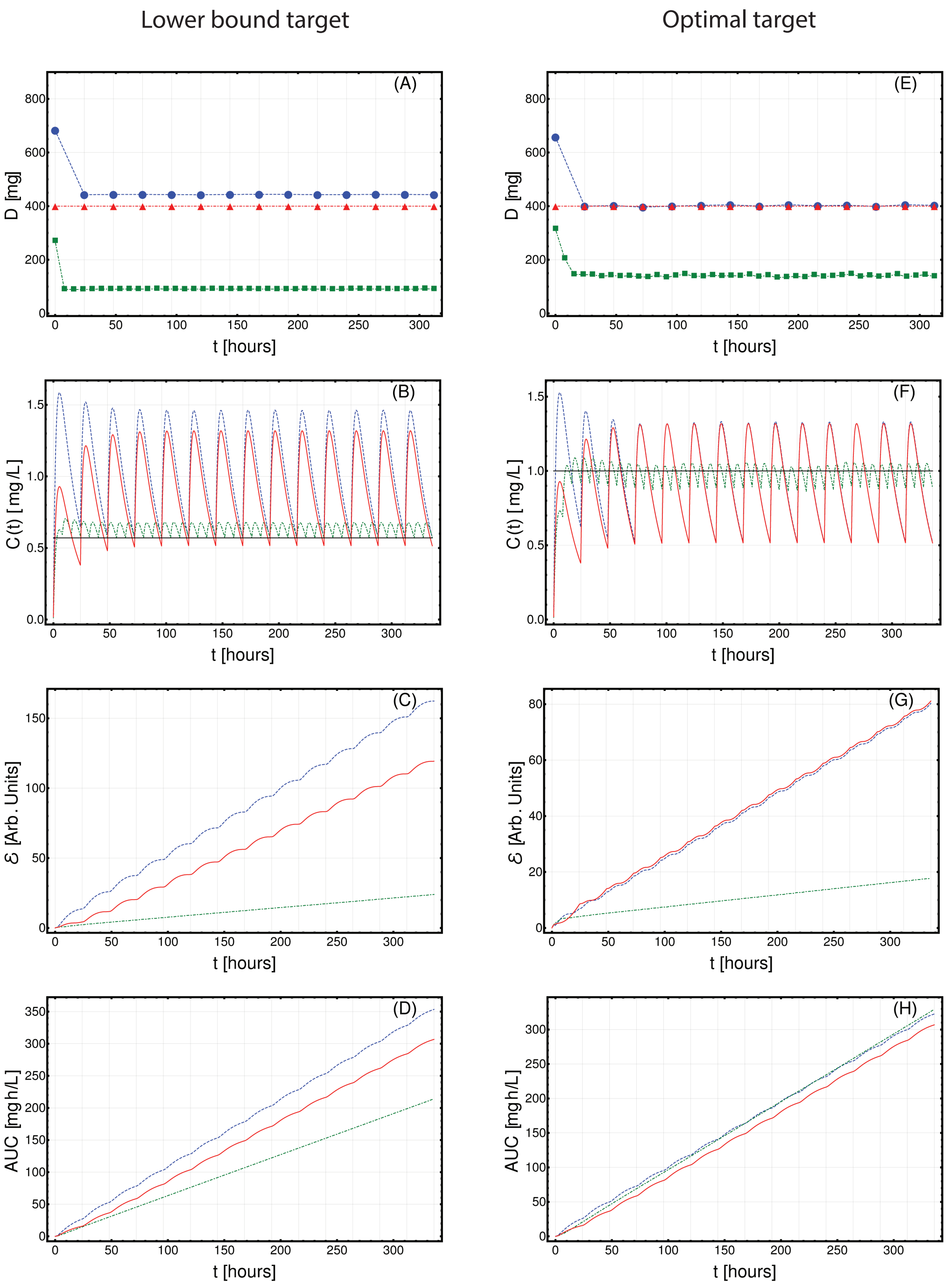

Figure S20: Optimized Imatinib administration returned by CT4TD for patient 0007 00002 DPS from (Michor et al. (2005)), in the cases of: 1-dose/day (blue) and 3-doses/day (green), with respect to: *lower-bound* target concentration $C_{targ} = 0.57\ [mg/L]$ (left panels: A–D) and *optimal* target concentration $C_{targ} = 1\ [mg/L]$ (right panels: E–H). Standard administration – i.e., 400 mg Imatinib/day – is shown with a red dashed line. In this case, the optimization of PK/PD models is obtained on patient-specific PK parameters, without considering the PD models. **(A,D)** Imatinib scheduled dosage in mg (y axis), displayed on 14 days (x axis). **(B-E)** Imatinib concentration in blood in $[mg/L]$ (y axis). **(C-F)** Variation of the cumulative distance between the observed concentration and the target in time.**(D-H)** The AUC in $[mg \cdot h/L]$

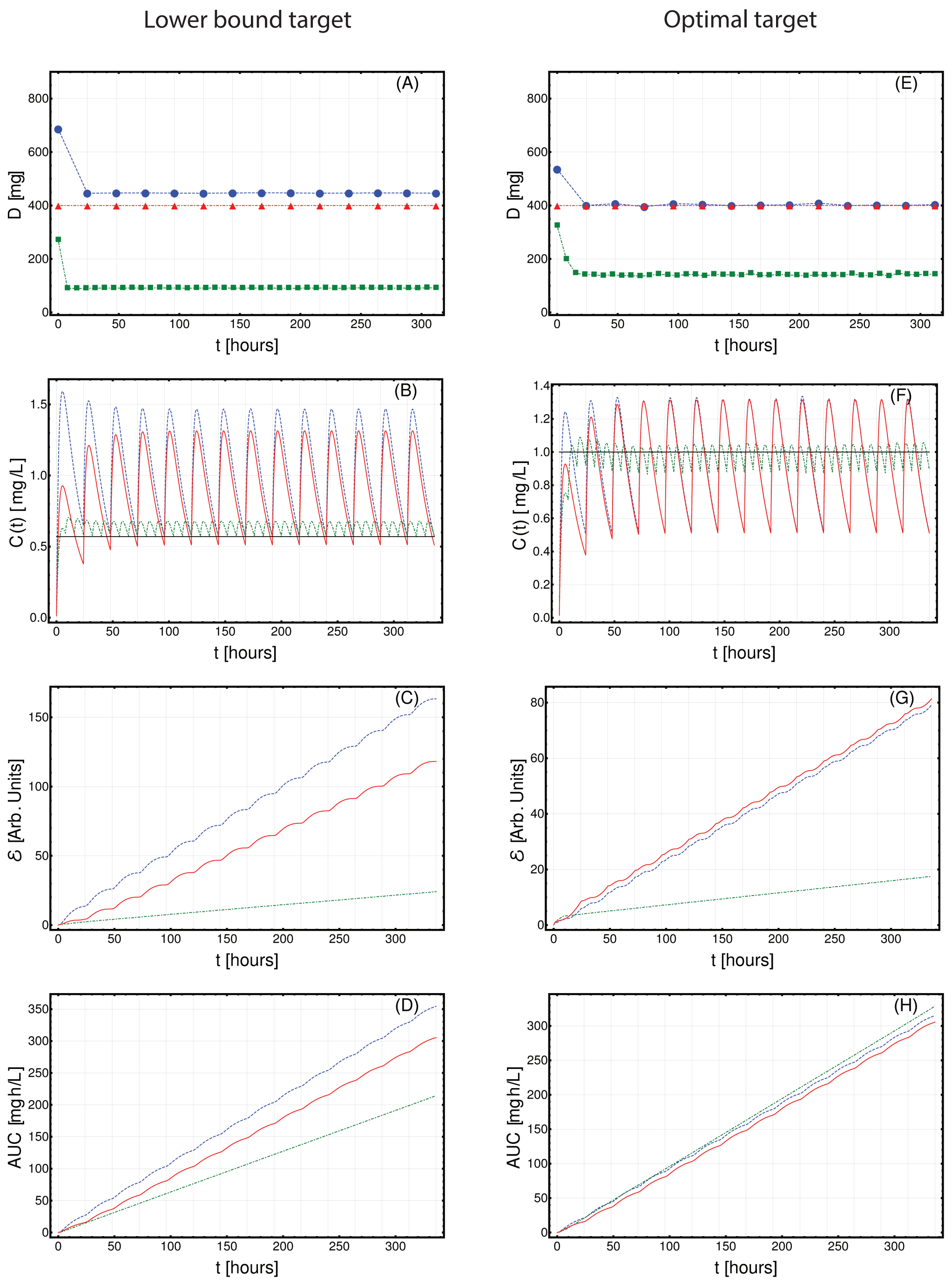

Figure S21: Optimized Imatinib administration returned by CT4TD for patient 0008 00002 PWR from (Michor et al. (2005)), in the cases of: 1-dose/day (blue) and 3-doses/day (green), with respect to: *lower-bound* target concentration $C_{targ} = 0.57\ [mg/L]$ (left panels: A–D) and *optimal* target concentration $C_{targ} = 1\ [mg/L]$ (right panels: E–H). Standard administration – i.e., 400 mg Imatinib/day – is shown with a red dashed line. In this case, the optimization of PK/PD models is obtained on patient-specific PK parameters, without considering the PD models. **(A,D)** Imatinib scheduled dosage in mg (y axis), displayed on 14 days (x axis). **(B-E)** Imatinib concentration in blood in $[mg/L]$ (y axis). **(C-F)** Variation of the cumulative distance between the observed concentration and the target in time.**(D-H)** The AUC in $[mg \cdot h/L]$

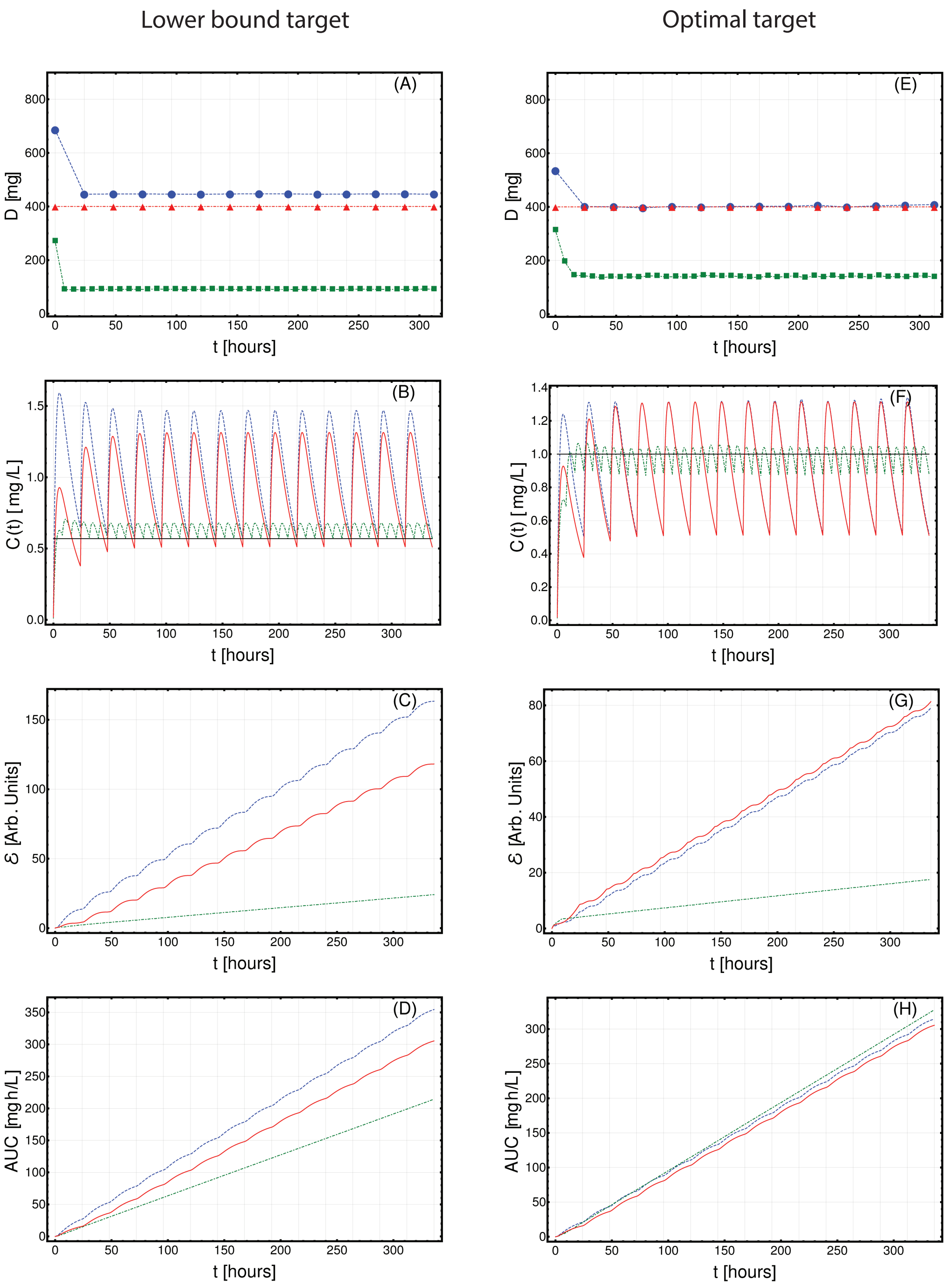

Figure S22: Optimized Imatinib administration returned by CT4TD for patient 0008 00003 LJG from (Michor et al. (2005)), in the cases of: 1-dose/day (blue) and 3-doses/day (green), with respect to: *lower-bound* target concentration $C_{targ} = 0.57\ [mg/L]$ (left panels: A–D) and *optimal* target concentration $C_{targ} = 1\ [mg/L]$ (right panels: E–H). Standard administration – i.e., 400 mg Imatinib/day – is shown with a red dashed line. In this case, the optimization of PK/PD models is obtained on patient-specific PK parameters, without considering the PD models. **(A,D)** Imatinib scheduled dosage in mg (y axis), displayed on 14 days (x axis). **(B-E)** Imatinib concentration in blood in $[mg/L]$ (y axis). **(C-F)** Variation of the cumulative distance between the observed concentration and the target in time.**(D-H)** The AUC in $[mg \cdot h/L]$

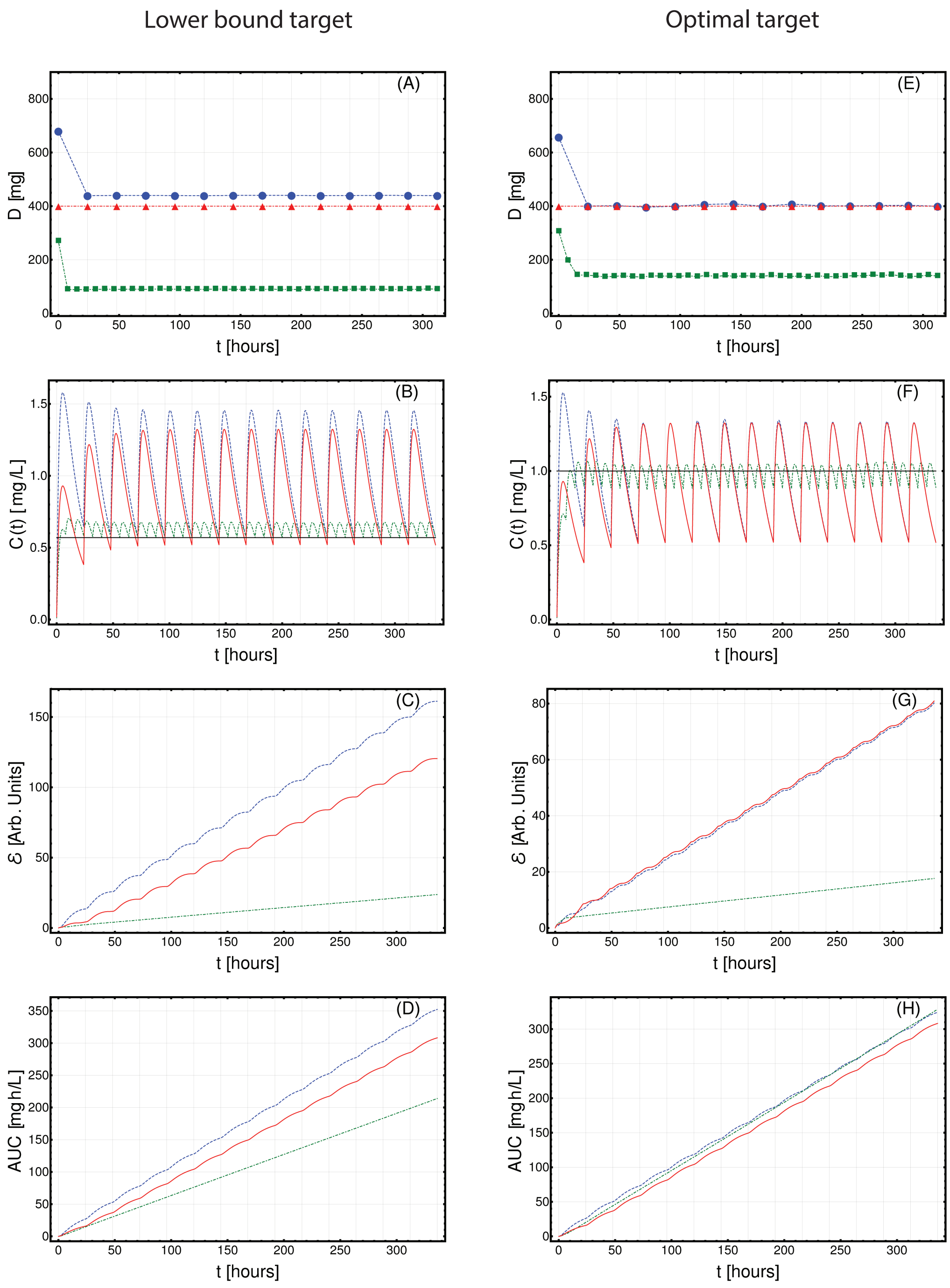

Figure S23: Optimized Imatinib administration returned by CT4TD for patient 0008 00005 BAT from (Michor et al. (2005)), in the cases of: 1-dose/day (blue) and 3-doses/day (green), with respect to: *lower-bound* target concentration $C_{targ} = 0.57\ [mg/L]$ (left panels: A–D) and *optimal* target concentration $C_{targ} = 1\ [mg/L]$ (right panels: E–H). Standard administration – i.e., 400 mg Imatinib/day – is shown with a red dashed line. In this case, the optimization of PK/PD models is obtained on patient-specific PK parameters, without considering the PD models. **(A,D)** Imatinib scheduled dosage in mg (y axis), displayed on 14 days (x axis). **(B-E)** Imatinib concentration in blood in $[mg/L]$ (y axis). **(C-F)** Variation of the cumulative distance between the observed concentration and the target in time.**(D-H)** The AUC in $[mg \cdot h/L]$

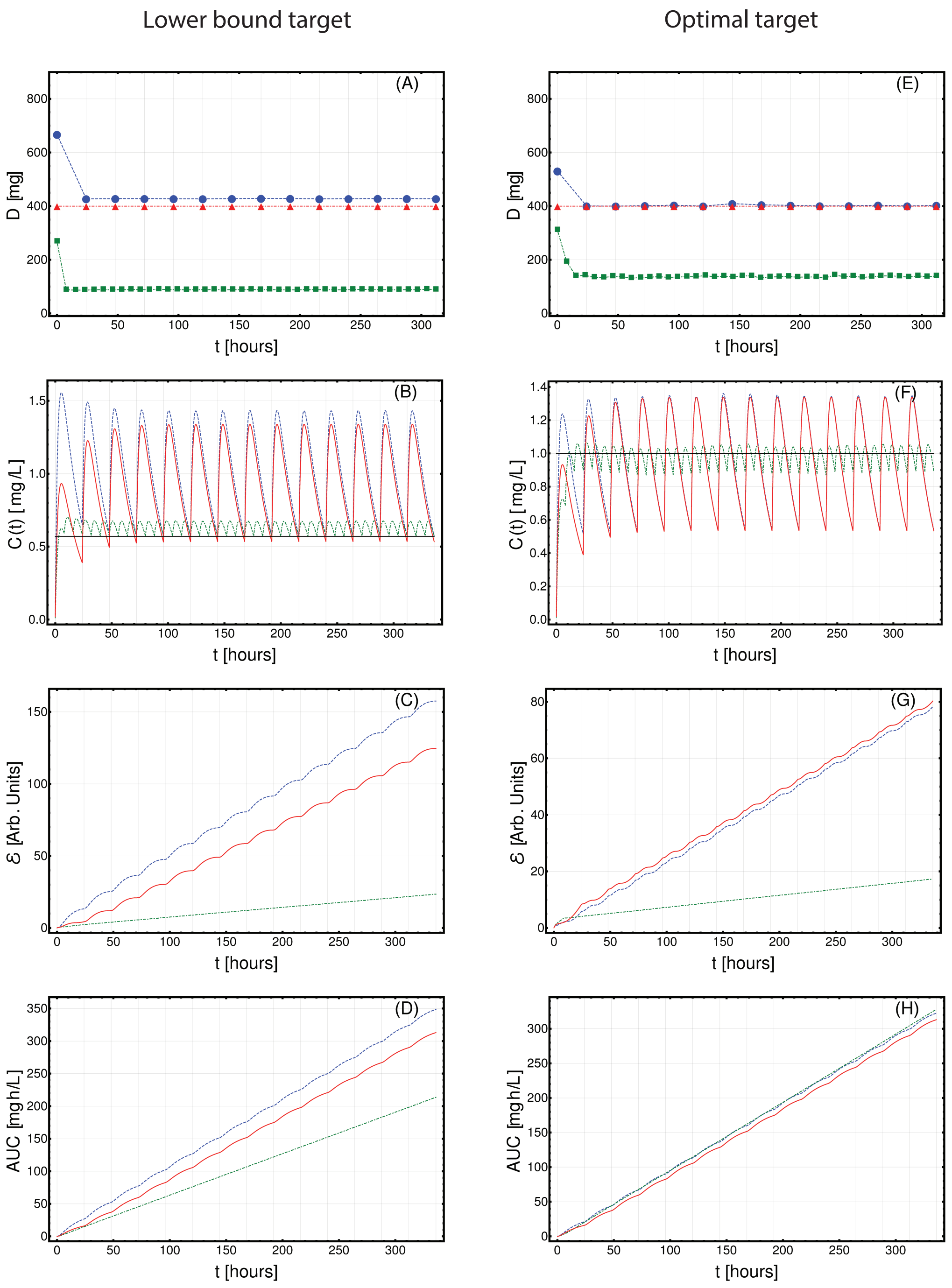

Figure S24: Optimized Imatinib administration returned by CT4TD for patient 0009 00003 LH from (Michor et al. (2005)), in the cases of: 1-dose/day (blue) and 3-doses/day (green), with respect to: *lower-bound* target concentration $C_{targ} = 0.57\ [mg/L]$ (left panels: A–D) and *optimal* target concentration $C_{targ} = 1\ [mg/L]$ (right panels: E–H). Standard administration – i.e., 400 mg Imatinib/day – is shown with a red dashed line. In this case, the optimization of PK/PD models is obtained on patient-specific PK parameters, without considering the PD models. **(A,D)** Imatinib scheduled dosage in mg (y axis), displayed on 14 days (x axis). **(B-E)** Imatinib concentration in blood in $[mg/L]$ (y axis). **(C-F)** Variation of the cumulative distance between the observed concentration and the target in time.**(D-H)** The AUC in $[mg \cdot h/L]$

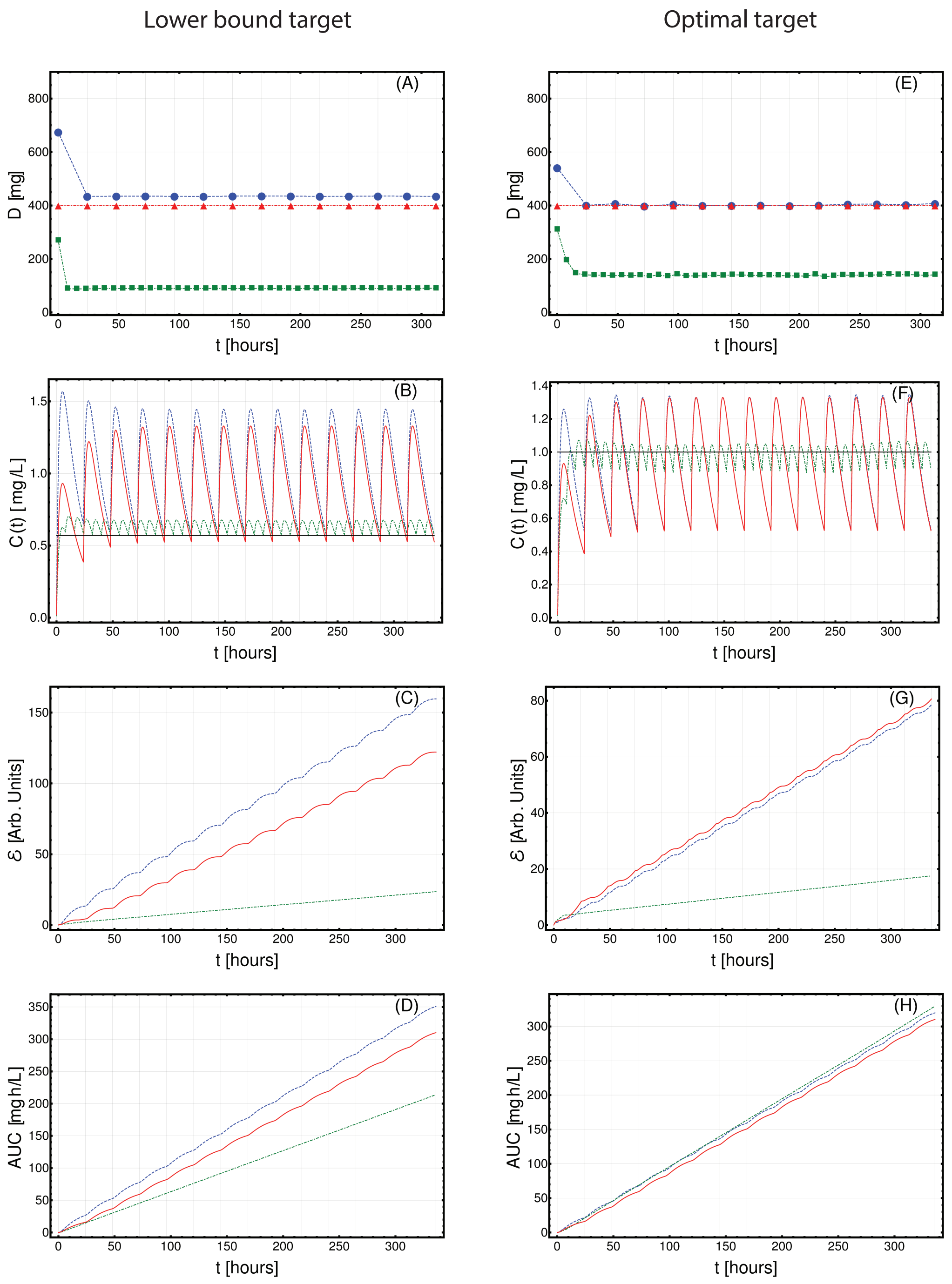

Figure S25: Optimized Imatinib administration returned by CT4TD for patient 0010 00001 HJ from (Michor et al. (2005)), in the cases of: 1-dose/day (blue) and 3-doses/day (green), with respect to: *lower-bound* target concentration $C_{targ} = 0.57\ [mg/L]$ (left panels: A–D) and *optimal* target concentration $C_{targ} = 1\ [mg/L]$ (right panels: E–H). Standard administration – i.e., 400 mg Imatinib/day – is shown with a red dashed line. In this case, the optimization of PK/PD models is obtained on patient-specific PK parameters, without considering the PD models. **(A,D)** Imatinib scheduled dosage in mg (y axis), displayed on 14 days (x axis). **(B-E)** Imatinib concentration in blood in $[mg/L]$ (y axis). **(C-F)** Variation of the cumulative distance between the observed concentration and the target in time.**(D-H)** The AUC in $[mg \cdot h/L]$

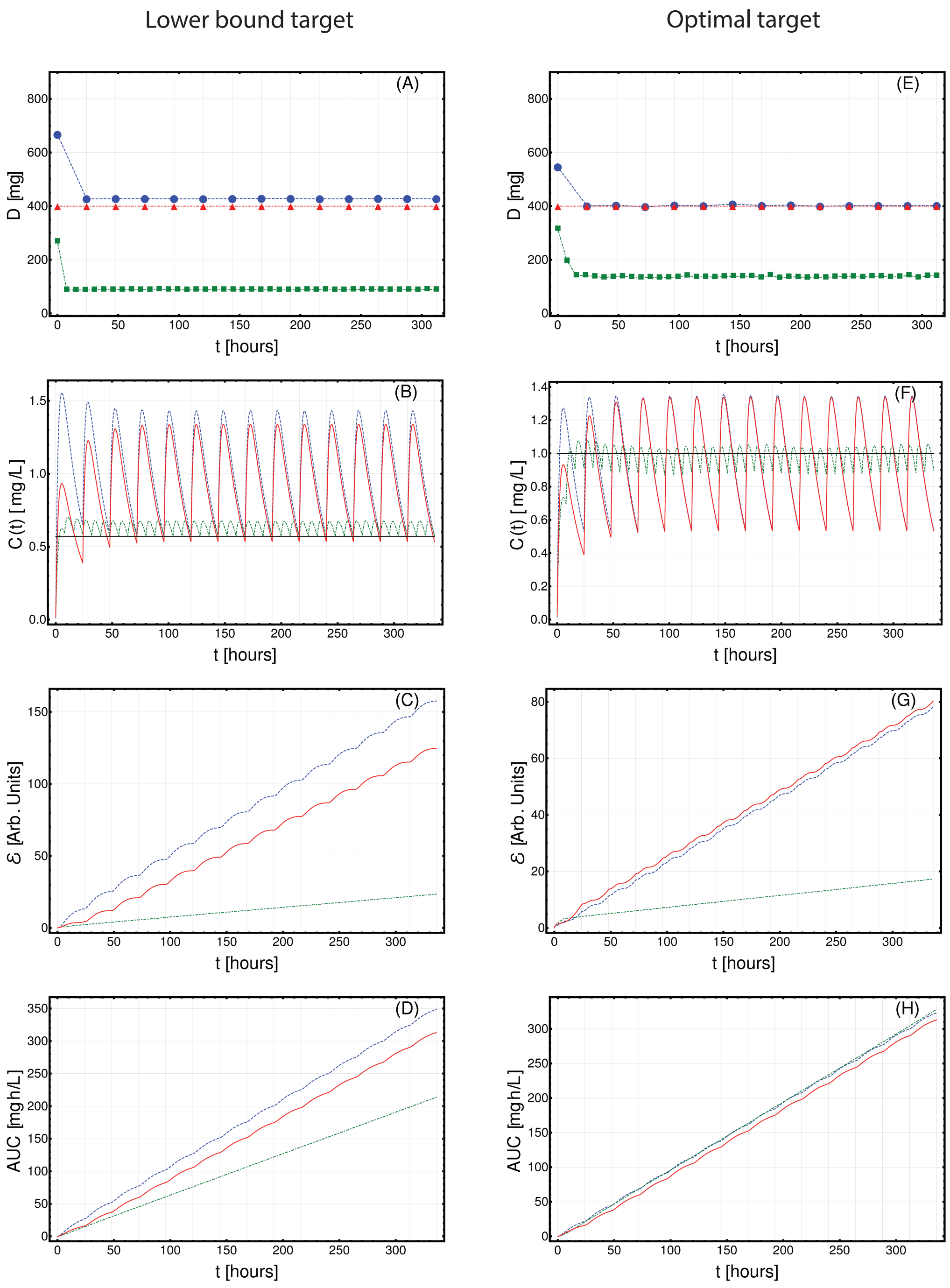

Figure S26: Optimized Imatinib administration returned by CT4TD for patient 0010 00002 GR from (Michor et al. (2005)), in the cases of: 1-dose/day (blue) and 3-doses/day (green), with respect to: *lower-bound* target concentration $C_{targ} = 0.57\ [mg/L]$ (left panels: A–D) and *optimal* target concentration $C_{targ} = 1\ [mg/L]$ (right panels: E–H). Standard administration – i.e., 400 mg Imatinib/day – is shown with a red dashed line. In this case, the optimization of PK/PD models is obtained on patient-specific PK parameters, without considering the PD models. **(A,D)** Imatinib scheduled dosage in mg (y axis), displayed on 14 days (x axis). **(B-E)** Imatinib concentration in blood in $[mg/L]$ (y axis). **(C-F)** Variation of the cumulative distance between the observed concentration and the target in time.**(D-H)** The AUC in $[mg \cdot h/L]$

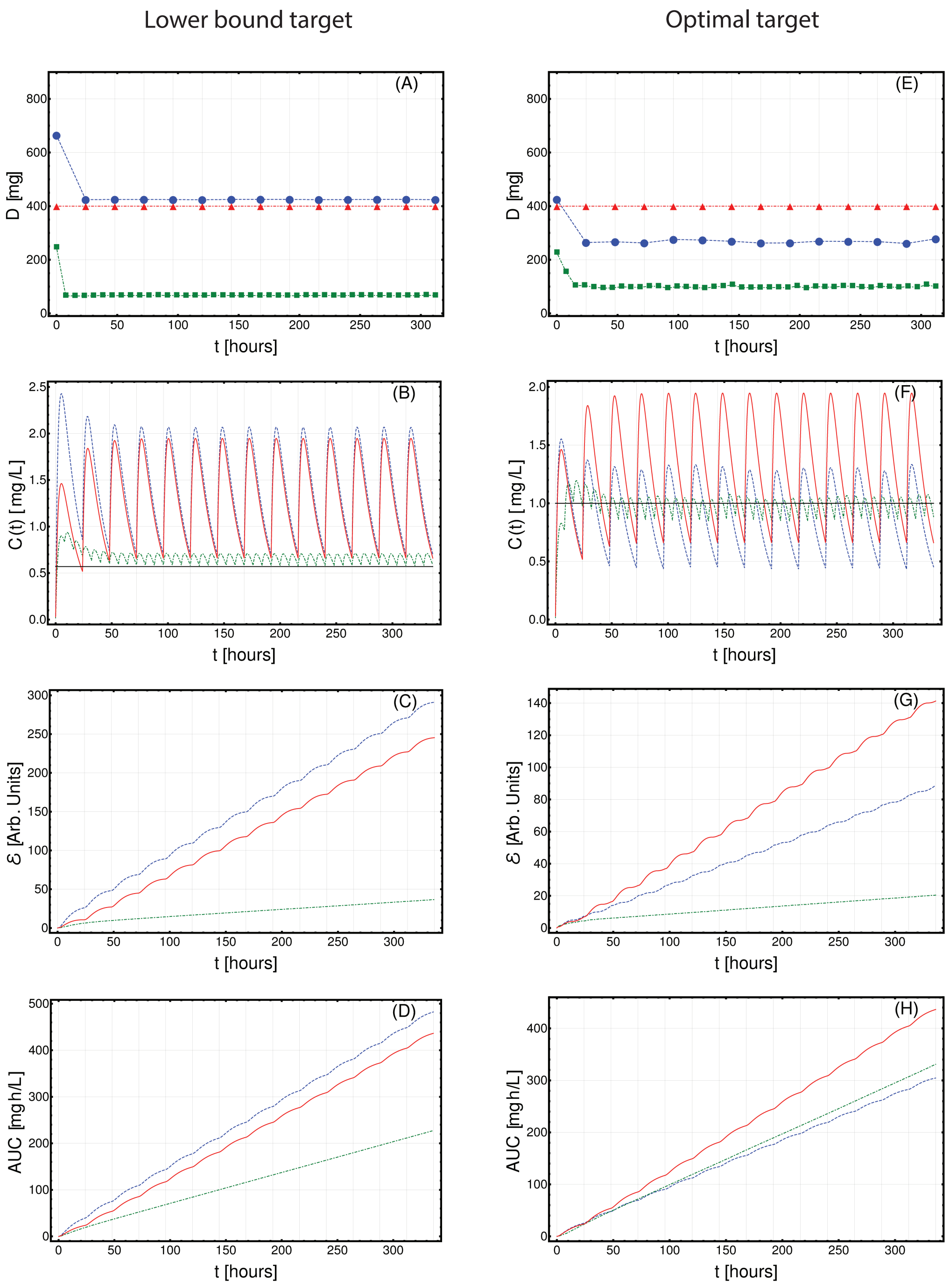

Figure S27: Optimized Imatinib administration returned by CT4TD for patient 0011 00001 CMC from (Michor et al. (2005)), in the cases of: 1-dose/day (blue) and 3-doses/day (green), with respect to: *lower-bound* target concentration $C_{targ} = 0.57\ [mg/L]$ (left panels: A–D) and *optimal* target concentration $C_{targ} = 1\ [mg/L]$ (right panels: E–H). Standard administration – i.e., 400 mg Imatinib/day – is shown with a red dashed line. In this case, the optimization of PK/PD models is obtained on patient-specific PK parameters, without considering the PD models. **(A,D)** Imatinib scheduled dosage in mg (y axis), displayed on 14 days (x axis). **(B-E)** Imatinib concentration in blood in $[mg/L]$ (y axis). **(C-F)** Variation of the cumulative distance between the observed concentration and the target in time.**(D-H)** The AUC in $[mg \cdot h/L]$

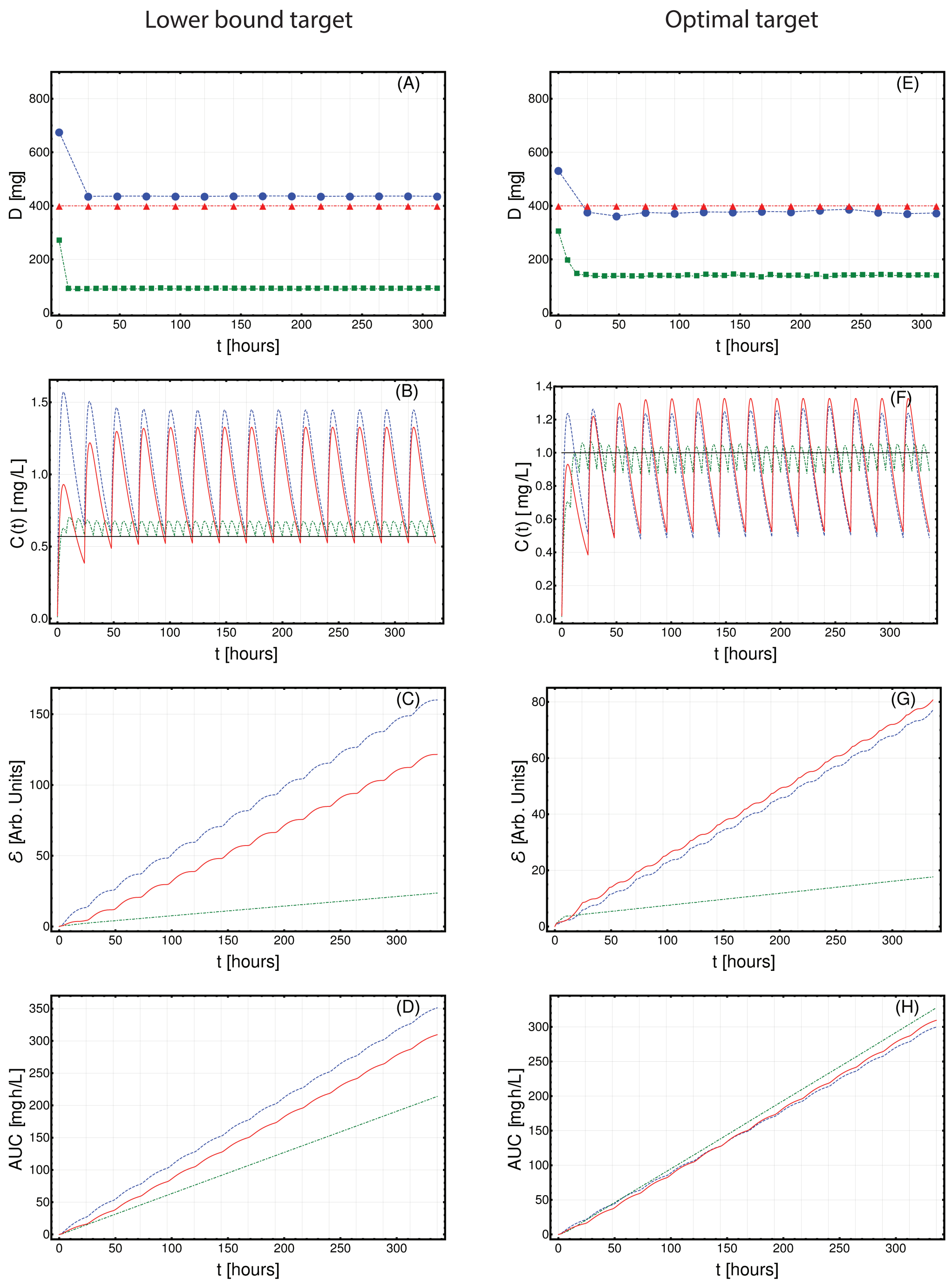

Figure S28: Optimized Imatinib administration returned by CT4TD for patient 0011 00005 SCW from (Michor et al. (2005)), in the cases of: 1-dose/day (blue) and 3-doses/day (green), with respect to: *lower-bound* target concentration $C_{targ} = 0.57\ [mg/L]$ (left panels: A–D) and *optimal* target concentration $C_{targ} = 1\ [mg/L]$ (right panels: E–H). Standard administration – i.e., 400 mg Imatinib/day – is shown with a red dashed line. In this case, the optimization of PK/PD models is obtained on patient-specific PK parameters, without considering the PD models. **(A,D)** Imatinib scheduled dosage in mg (y axis), displayed on 14 days (x axis). **(B-E)** Imatinib concentration in blood in $[mg/L]$ (y axis). **(C-F)** Variation of the cumulative distance between the observed concentration and the target in time.**(D-H)** The AUC in $[mg \cdot h/L]$

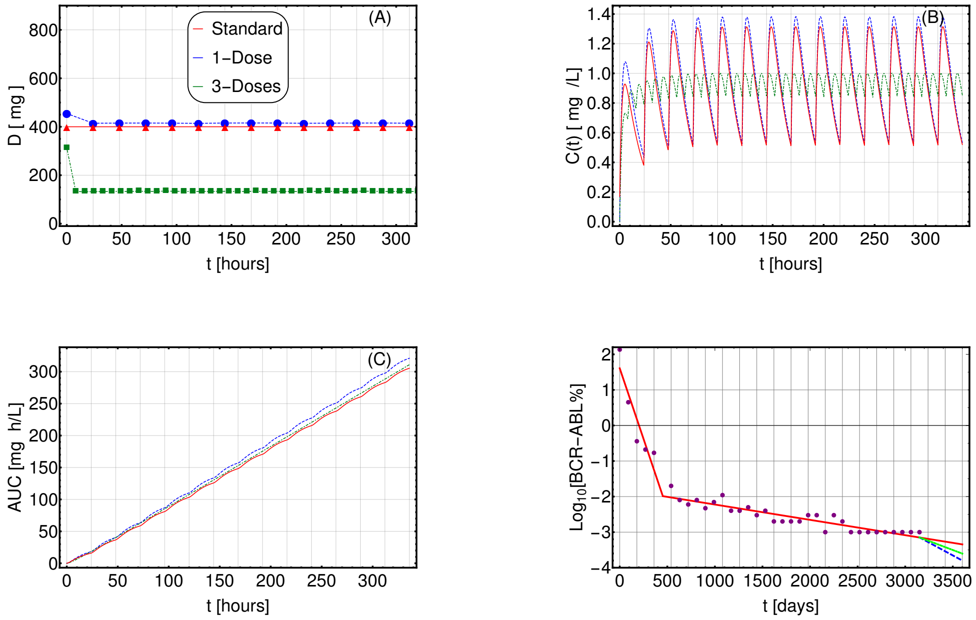

Figure S29: **Adjusting therapy for tumor burden minimization.** Imatinib administration optimized for tumor burden minimization in patient 0001 00002 RH (male) from (Michor et al. (2005)), in the cases of: 1-dose/day (purple) and 3-doses/day (green), with $\phi = 60$, as compared to standard administration (red). In this case, the optimization is obtained on patient-specific PK and PD models. **(A)** Imatinib scheduled dosage in mg (y axis), displayed on 14 days (x axis). **(B)** Imatinib concentration in blood in $[mg/L]$ (y axis). **(C)** Temporal variation of the AUC in $[mg \cdot h/L]$ (y axis). **(D)** Longitudinal data points on tumor burden recorded are presented in purple. The best fit is shown with red lines. The slope of the right-most line is used to determine the cancer stem cell death rate and, in turn, the patient-specific PD parameters. The blue (green) line represents the predicted cancer subpopulation decay in case the 1-dose (3-doses) optimal administration

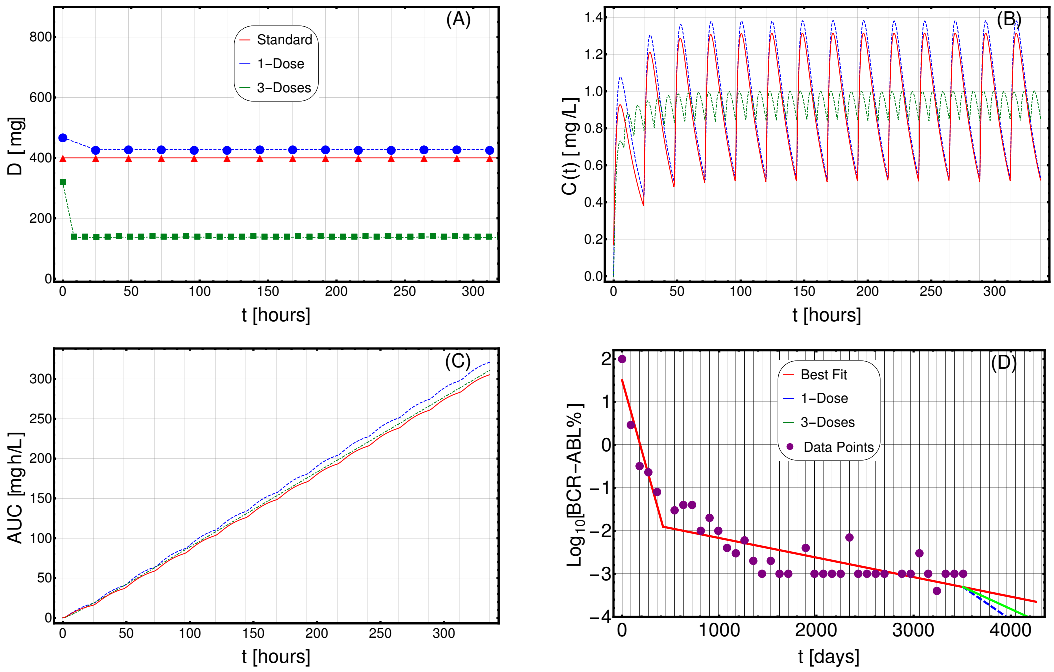

Figure S30: **Adjusting therapy for tumor burden minimization.** Imatinib administration optimized for tumor burden minimization in patient 0001 00006 GMC (male) from (Michor et al. (2005)), in the cases of: 1-dose/day (purple) and 3-doses/day (green), with $\phi = 60$, as compared to standard administration (red). In this case, the optimization is obtained on patient-specific PK and PD models. **(A)** Imatinib scheduled dosage in mg (y axis), displayed on 14 days (x axis). **(B)** Imatinib concentration in blood in $[mg/L]$ (y axis). **(C)** Temporal variation of the AUC in $[mg \cdot h/L]$ (y axis). **(D)** Longitudinal data points on tumor burden recorded are presented in purple. The best fit is shown with red lines. The slope of the right-most line is used to determine the cancer stem cell death rate and, in turn, the patient-specific PD parameters. The blue (green) line represents the predicted cancer subpopulation decay in case the 1-dose (3-doses) optimal administration

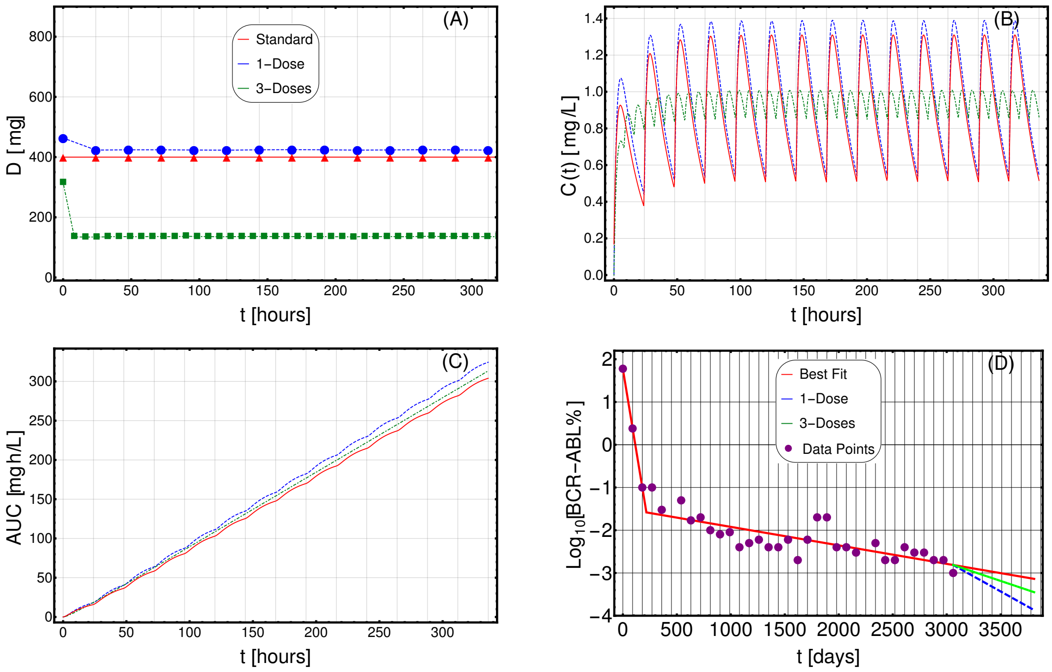

Figure S31: **Adjusting therapy for tumor burden minimization.** Imatinib administration optimized for tumor burden minimization in patient 0001 00009 MJG (male) from (Michor et al. (2005)), in the cases of: 1-dose/day (purple) and 3-doses/day (green), with $\phi = 60$, as compared to standard administration (red). In this case, the optimization is obtained on patient-specific PK and PD models. **(A)** Imatinib scheduled dosage in mg (y axis), displayed on 14 days (x axis). **(B)** Imatinib concentration in blood in $[mg/L]$ (y axis). **(C)** Temporal variation of the AUC in $[mg \cdot h/L]$ (y axis). **(D)** Longitudinal data points on tumor burden recorded are presented in purple. The best fit is shown with red lines. The slope of the right-most line is used to determine the cancer stem cell death rate and, in turn, the patient-specific PD parameters. The blue (green) line represents the predicted cancer subpopulation decay in case the 1-dose (3-doses) optimal administration

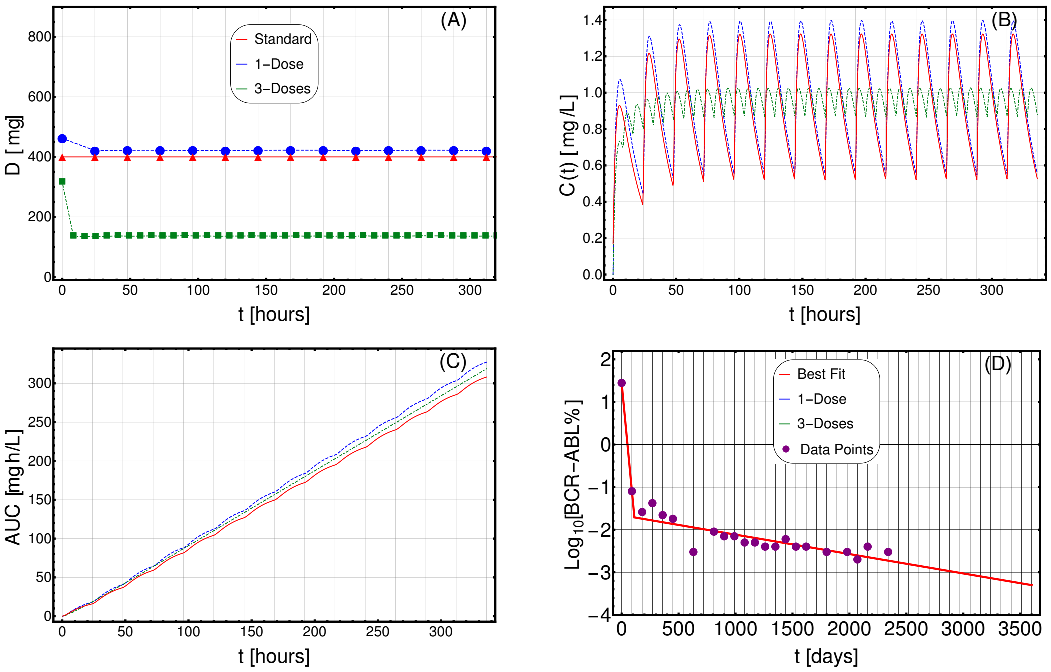

Figure S32: **Adjusting therapy for tumor burden minimization.** Imatinib administration optimized for tumor burden minimization in patient 0002 00003 SMV (male) from (Michor et al. (2005)), in the cases of: 1-dose/day (purple) and 3-doses/day (green), with $\phi = 60$, as compared to standard administration (red). In this case, the optimization is obtained on patient-specific PK and PD models. **(A)** Imatinib scheduled dosage in mg (y axis), displayed on 14 days (x axis). **(B)** Imatinib concentration in blood in $[mg/L]$ (y axis). **(C)** Temporal variation of the AUC in $[mg \cdot h/L]$ (y axis). **(D)** Longitudinal data points on tumor burden recorded are presented in purple. The best fit is shown with red lines. The slope of the right-most line is used to determine the cancer stem cell death rate and, in turn, the patient-specific PD parameters. The blue (green) line represents the predicted cancer subpopulation decay in case the 1-dose (3-doses) optimal administration

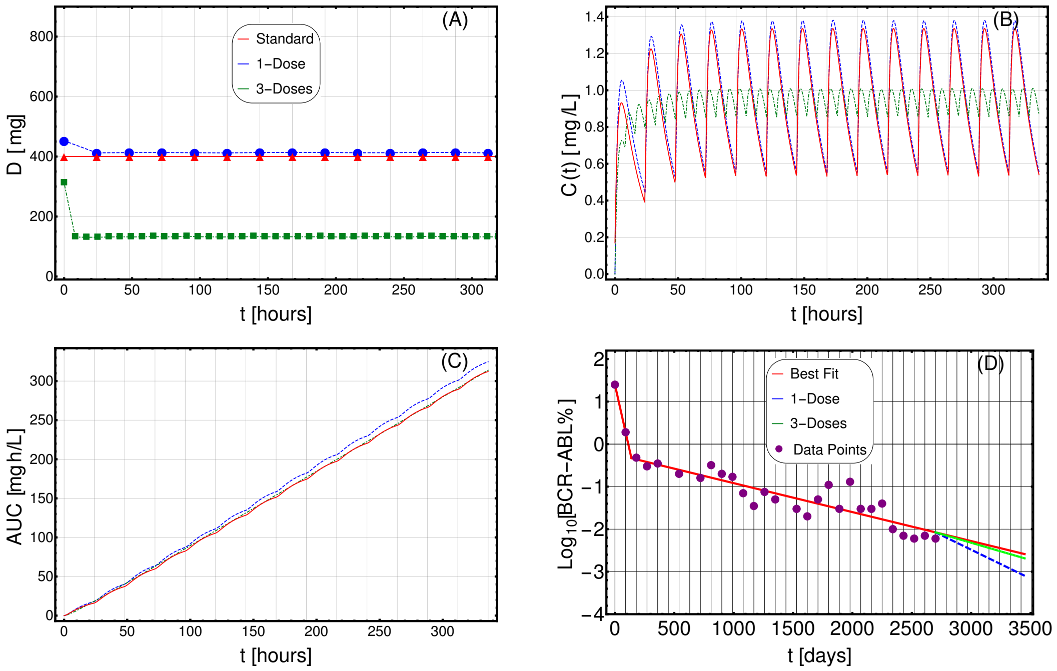

Figure S33: **Adjusting therapy for tumor burden minimization.** Imatinib administration optimized for tumor burden minimization in patient 0002 00007 PMG (male) from (Michor et al. (2005)), in the cases of: 1-dose/day (purple) and 3-doses/day (green), with $\phi = 60$, as compared to standard administration (red). In this case, the optimization is obtained on patient-specific PK and PD models. **(A)** Imatinib scheduled dosage in mg (y axis), displayed on 14 days (x axis). **(B)** Imatinib concentration in blood in $[mg/L]$ (y axis). **(C)** Temporal variation of the AUC in $[mg \cdot h/L]$ (y axis). **(D)** Longitudinal data points on tumor burden recorded are presented in purple. The best fit is shown with red lines. The slope of the right-most line is used to determine the cancer stem cell death rate and, in turn, the patient-specific PD parameters. The blue (green) line represents the predicted cancer subpopulation decay in case the 1-dose (3-doses) optimal administration

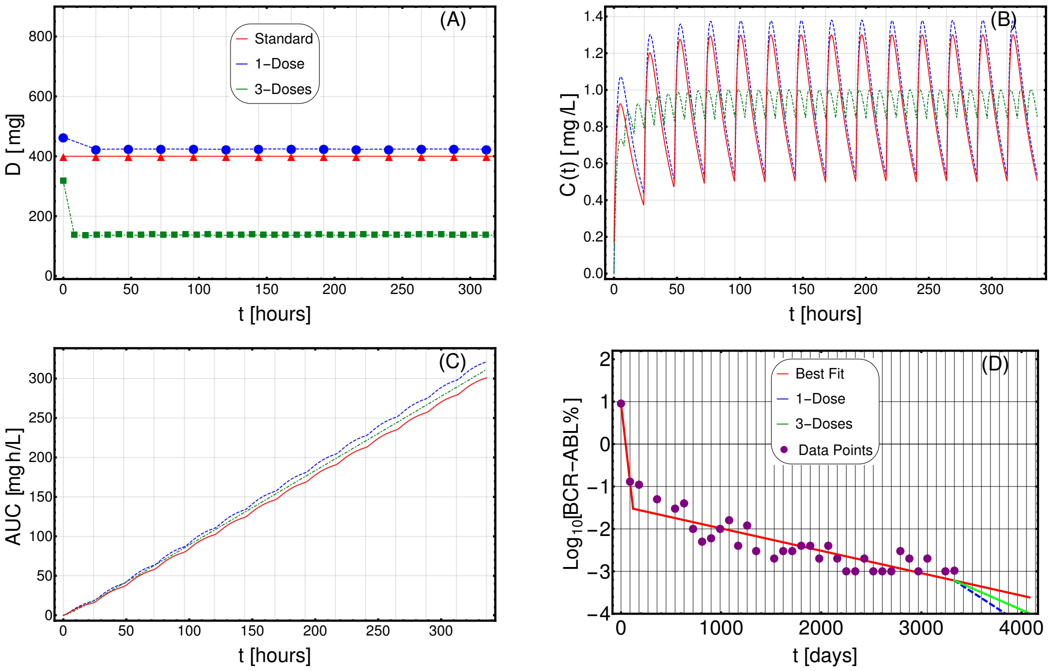

Figure S34: **Adjusting therapy for tumor burden minimization.** Imatinib administration optimized for tumor burden minimization in patient 0002 00008 SAT (male) from (Michor et al. (2005)), in the cases of: 1-dose/day (purple) and 3-doses/day (green), with $\phi = 60$, as compared to standard administration (red). In this case, the optimization is obtained on patient-specific PK and PD models. **(A)** Imatinib scheduled dosage in mg (y axis), displayed on 14 days (x axis). **(B)** Imatinib concentration in blood in $[mg/L]$ (y axis). **(C)** Temporal variation of the AUC in $[mg \cdot h/L]$ (y axis). **(D)** Longitudinal data points on tumor burden recorded are presented in purple. The best fit is shown with red lines. The slope of the right-most line is used to determine the cancer stem cell death rate and, in turn, the patient-specific PD parameters. The blue (green) line represents the predicted cancer subpopulation decay in case the 1-dose (3-doses) optimal administration

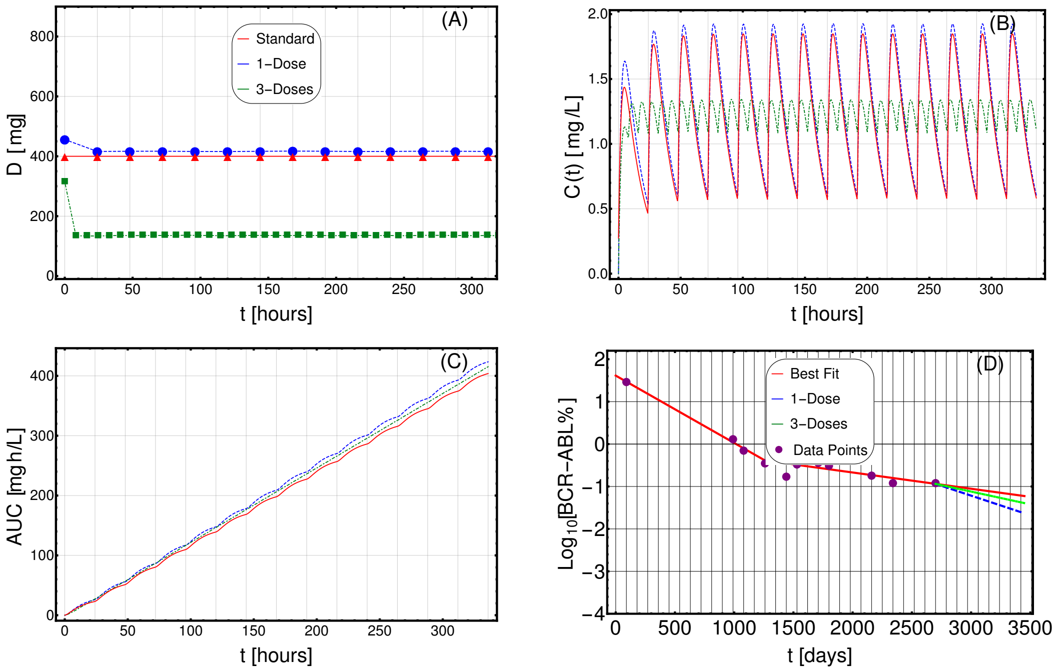

Figure S35: **Adjusting therapy for tumor burden minimization.** Imatinib administration optimized for tumor burden minimization in patient 0002 00008 CL (female) from (Michor et al. (2005)), in the cases of: 1-dose/day (purple) and 3-doses/day (green), with $\phi = 75$, as compared to standard administration (red). In this case, the optimization is obtained on patient-specific PK and PD models. **(A)** Imatinib scheduled dosage in mg (y axis), displayed on 14 days (x axis). **(B)** Imatinib concentration in blood in $[mg/L]$ (y axis). **(C)** Temporal variation of the AUC in $[mg \cdot h/L]$ (y axis). **(D)** Longitudinal data points on tumor burden recorded are presented in purple. The best fit is shown with red lines. The slope of the right-most line is used to determine the cancer stem cell death rate and, in turn, the patient-specific PD parameters. The blue (green) line represents the predicted cancer subpopulation decay in case the 1-dose (3-doses) optimal administration

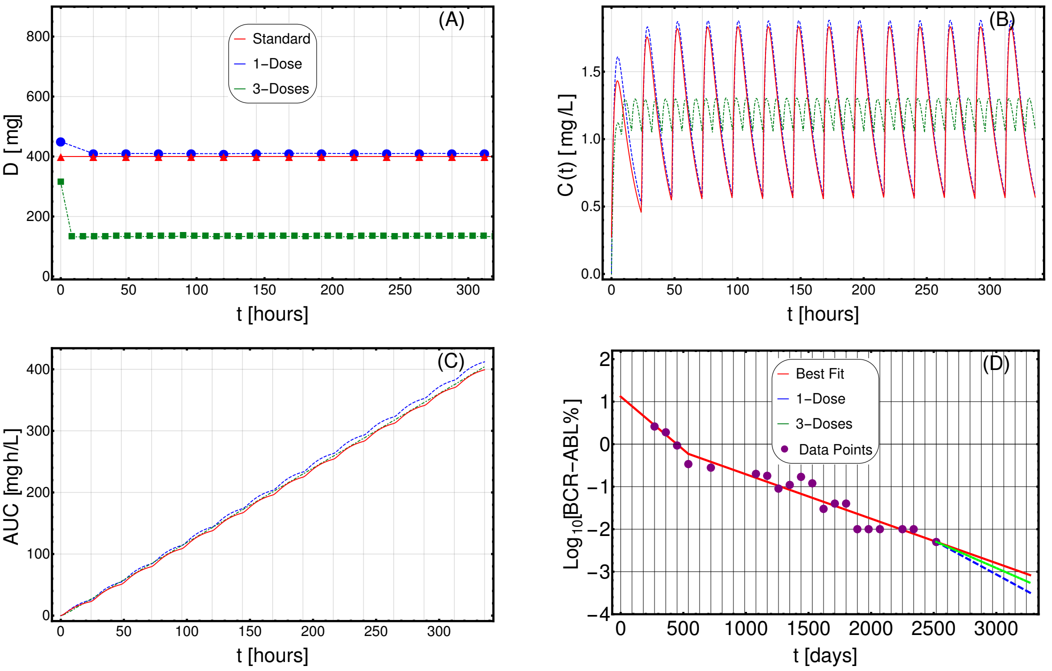

Figure S36: **Adjusting therapy for tumor burden minimization.** Imatinib administration optimized for tumor burden minimization in patient 0004 00003 CAR (male) from (Michor et al. (2005)), in the cases of: 1-dose/day (purple) and 3-doses/day (green), with $\phi = 75$, as compared to standard administration (red). In this case, the optimization is obtained on patient-specific PK and PD models. **(A)** Imatinib scheduled dosage in mg (y axis), displayed on 14 days (x axis). **(B)** Imatinib concentration in blood in $[mg/L]$ (y axis). **(C)** Temporal variation of the AUC in $[mg \cdot h/L]$ (y axis). **(D)** Longitudinal data points on tumor burden recorded are presented in purple. The best fit is shown with red lines. The slope of the right-most line is used to determine the cancer stem cell death rate and, in turn, the patient-specific PD parameters. The blue (green) line represents the predicted cancer subpopulation decay in case the 1-dose (3-doses) optimal administration

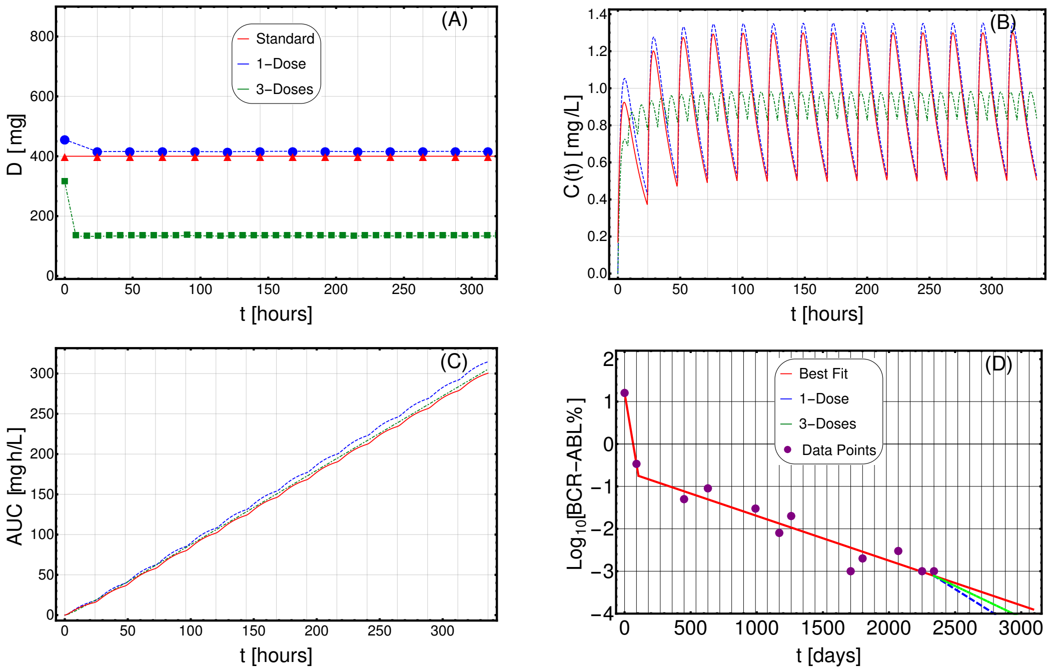

Figure S37: **Adjusting therapy for tumor burden minimization.** Imatinib administration optimized for tumor burden minimization in patient 0004 00006 GM (male) from (Michor et al. (2005)), in the cases of: 1-dose/day (purple) and 3-doses/day (green), with $\phi = 60$, as compared to standard administration (red). In this case, the optimization is obtained on patient-specific PK and PD models. **(A)** Imatinib scheduled dosage in mg (y axis), displayed on 14 days (x axis). **(B)** Imatinib concentration in blood in $[mg/L]$ (y axis). **(C)** Temporal variation of the AUC in $[mg \cdot h/L]$ (y axis). **(D)** Longitudinal data points on tumor burden recorded are presented in purple. The best fit is shown with red lines. The slope of the right-most line is used to determine the cancer stem cell death rate and, in turn, the patient-specific PD parameters. The blue (green) line represents the predicted cancer subpopulation decay in case the 1-dose (3-doses) optimal administration

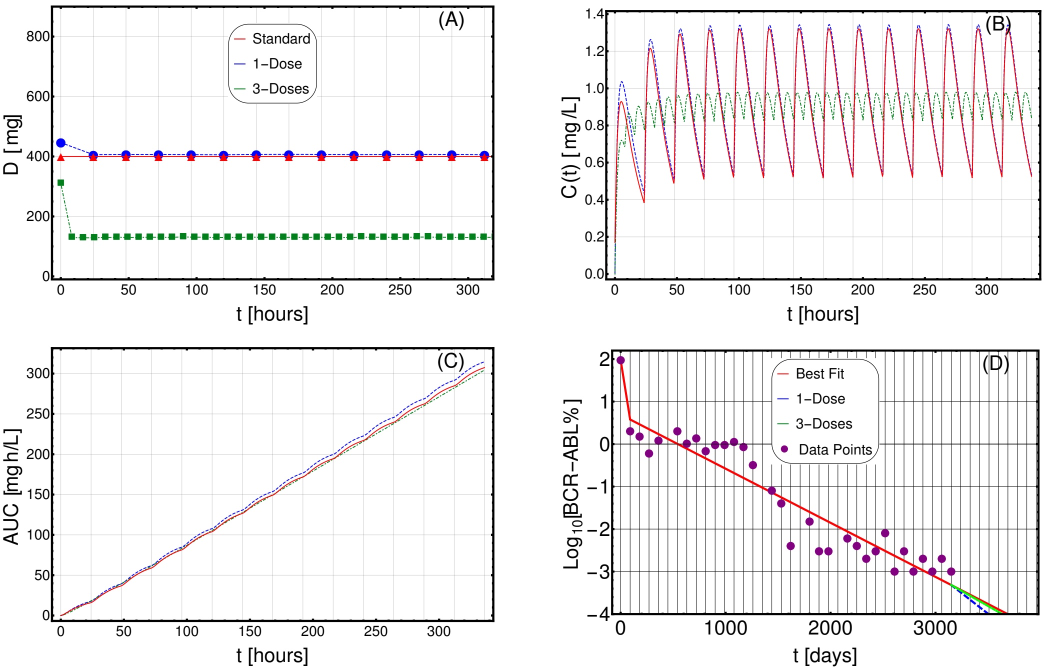

Figure S38: **Adjusting therapy for tumor burden minimization.** Imatinib administration optimized for tumor burden minimization in patient 0006 00006 ML (male) from (Michor et al. (2005)), in the cases of: 1-dose/day (purple) and 3-doses/day (green), with $\phi = 60$, as compared to standard administration (red). In this case, the optimization is obtained on patient-specific PK and PD models. **(A)** Imatinib scheduled dosage in mg (y axis), displayed on 14 days (x axis). **(B)** Imatinib concentration in blood in $[mg/L]$ (y axis). **(C)** Temporal variation of the AUC in $[mg \cdot h/L]$ (y axis). **(D)** Longitudinal data points on tumor burden recorded are presented in purple. The best fit is shown with red lines. The slope of the right-most line is used to determine the cancer stem cell death rate and, in turn, the patient-specific PD parameters. The blue (green) line represents the predicted cancer subpopulation decay in case the 1-dose (3-doses) optimal administration

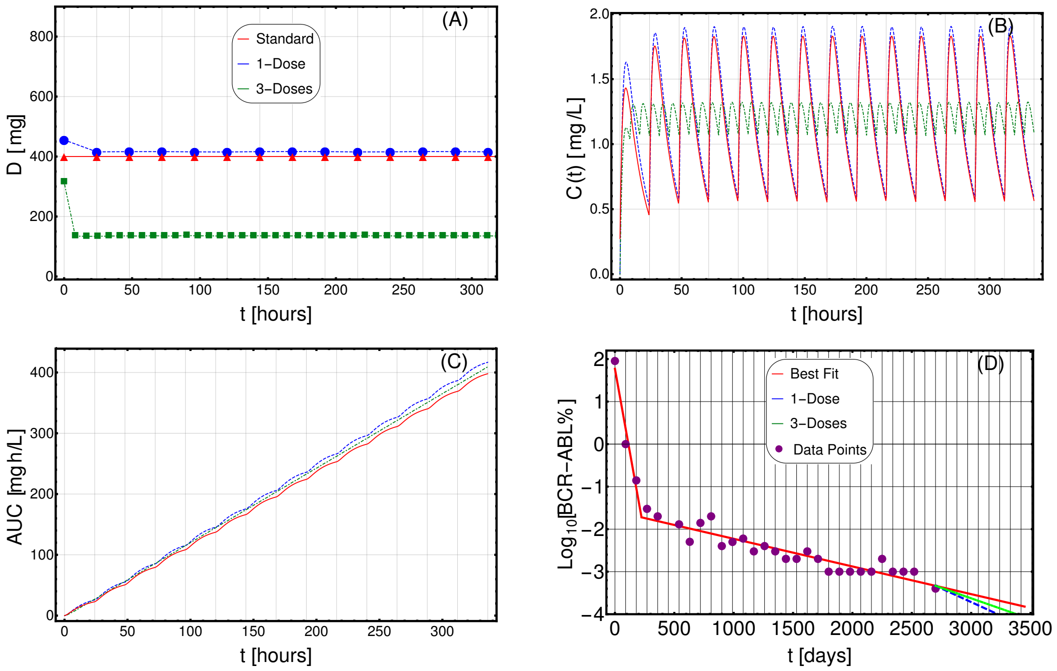

Figure S39: **Adjusting therapy for tumor burden minimization.** Imatinib administration optimized for tumor burden minimization in patient 0006 00007 RJW (female) from (Michor et al. (2005)), in the cases of: 1-dose/day (purple) and 3-doses/day (green), with $\phi = 75$, as compared to standard administration (red). In this case, the optimization is obtained on patient-specific PK and PD models. **(A)** Imatinib scheduled dosage in mg (y axis), displayed on 14 days (x axis). **(B)** Imatinib concentration in blood in $[mg/L]$ (y axis). **(C)** Temporal variation of the AUC in $[mg \cdot h/L]$ (y axis). **(D)** Longitudinal data points on tumor burden recorded are presented in purple. The best fit is shown with red lines. The slope of the right-most line is used to determine the cancer stem cell death rate and, in turn, the patient-specific PD parameters. The blue (green) line represents the predicted cancer subpopulation decay in case the 1-dose (3-doses) optimal administration

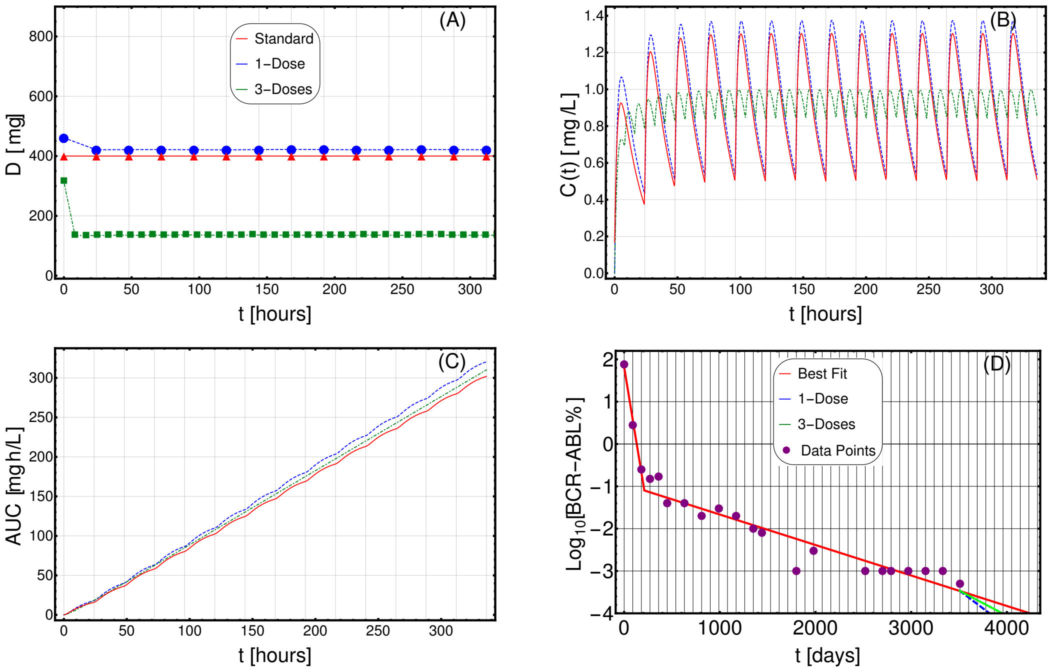

Figure S40: **Adjusting therapy for tumor burden minimization.** Imatinib administration optimized for tumor burden minimization in patient 0007 00001 AJV (male) from (Michor et al. (2005)), in the cases of: 1-dose/day (purple) and 3-doses/day (green), with $\phi = 60$, as compared to standard administration (red). In this case, the optimization is obtained on patient-specific PK and PD models. **(A)** Imatinib scheduled dosage in mg (y axis), displayed on 14 days (x axis). **(B)** Imatinib concentration in blood in $[mg/L]$ (y axis). **(C)** Temporal variation of the AUC in $[mg \cdot h/L]$ (y axis). **(D)** Longitudinal data points on tumor burden recorded are presented in purple. The best fit is shown with red lines. The slope of the right-most line is used to determine the cancer stem cell death rate and, in turn, the patient-specific PD parameters. The blue (green) line represents the predicted cancer subpopulation decay in case the 1-dose (3-doses) optimal administration

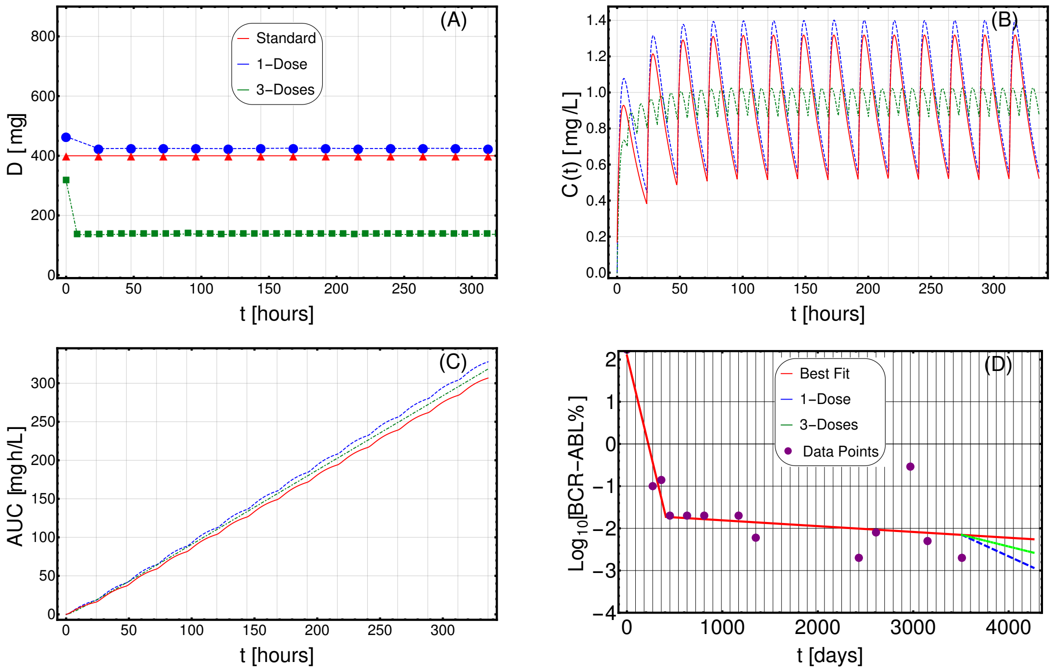

Figure S41: **Adjusting therapy for tumor burden minimization.** Imatinib administration optimized for tumor burden minimization in patient 0007 00002 DPS (male) from (Michor et al. (2005)), in the cases of: 1-dose/day (purple) and 3-doses/day (green), with $\phi = 60$, as compared to standard administration (red). In this case, the optimization is obtained on patient-specific PK and PD models. **(A)** Imatinib scheduled dosage in mg (y axis), displayed on 14 days (x axis). **(B)** Imatinib concentration in blood in $[mg/L]$ (y axis). **(C)** Temporal variation of the AUC in $[mg \cdot h/L]$ (y axis). **(D)** Longitudinal data points on tumor burden recorded are presented in purple. The best fit is shown with red lines. The slope of the right-most line is used to determine the cancer stem cell death rate and, in turn, the patient-specific PD parameters. The blue (green) line represents the predicted cancer subpopulation decay in case the 1-dose (3-doses) optimal administration

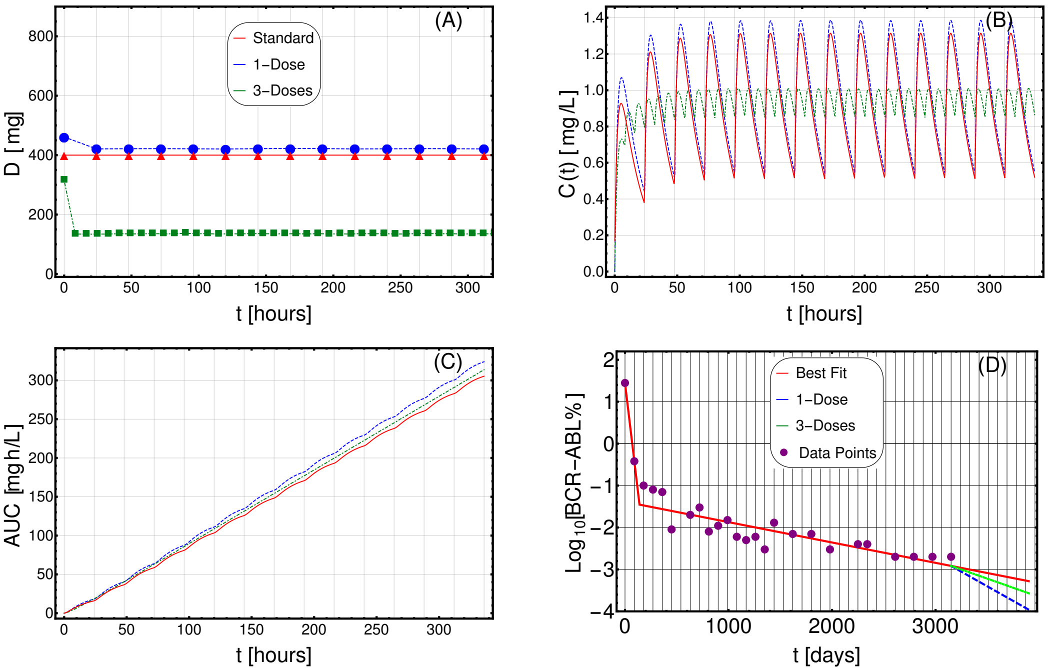

Figure S42: **Adjusting therapy for tumor burden minimization.** Imatinib administration optimized for tumor burden minimization in patient 0008 00002 PWR (male) from (Michor et al. (2005)), in the cases of: 1-dose/day (purple) and 3-doses/day (green), with $\phi = 60$, as compared to standard administration (red). In this case, the optimization is obtained on patient-specific PK and PD models. **(A)** Imatinib scheduled dosage in mg (y axis), displayed on 14 days (x axis). **(B)** Imatinib concentration in blood in $[mg/L]$ (y axis). **(C)** Temporal variation of the AUC in $[mg \cdot h/L]$ (y axis). **(D)** Longitudinal data points on tumor burden recorded are presented in purple. The best fit is shown with red lines. The slope of the right-most line is used to determine the cancer stem cell death rate and, in turn, the patient-specific PD parameters. The blue (green) line represents the predicted cancer subpopulation decay in case the 1-dose (3-doses) optimal administration

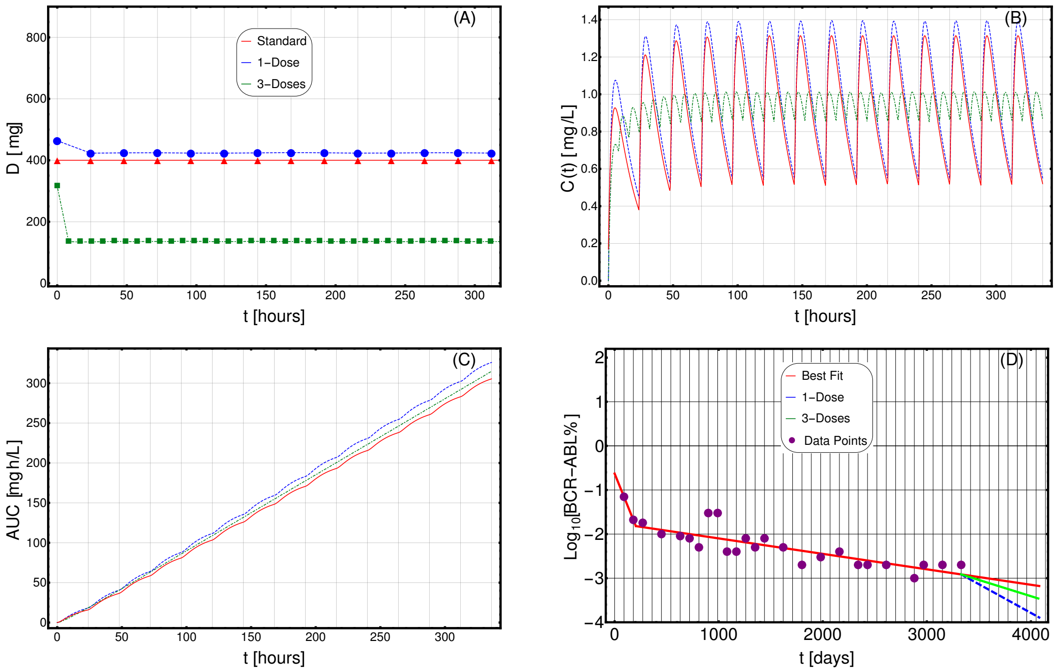

Figure S43: **Adjusting therapy for tumor burden minimization.** Imatinib administration optimized for tumor burden minimization in patient 0008 00003 LJG (male) from (Michor et al. (2005)), in the cases of: 1-dose/day (purple) and 3-doses/day (green), with $\phi = 60$, as compared to standard administration (red). In this case, the optimization is obtained on patient-specific PK and PD models. **(A)** Imatinib scheduled dosage in mg (y axis), displayed on 14 days (x axis). **(B)** Imatinib concentration in blood in $[mg/L]$ (y axis). **(C)** Temporal variation of the AUC in $[mg \cdot h/L]$ (y axis). **(D)** Longitudinal data points on tumor burden recorded are presented in purple. The best fit is shown with red lines. The slope of the right-most line is used to determine the cancer stem cell death rate and, in turn, the patient-specific PD parameters. The blue (green) line represents the predicted cancer subpopulation decay in case the 1-dose (3-doses) optimal administration

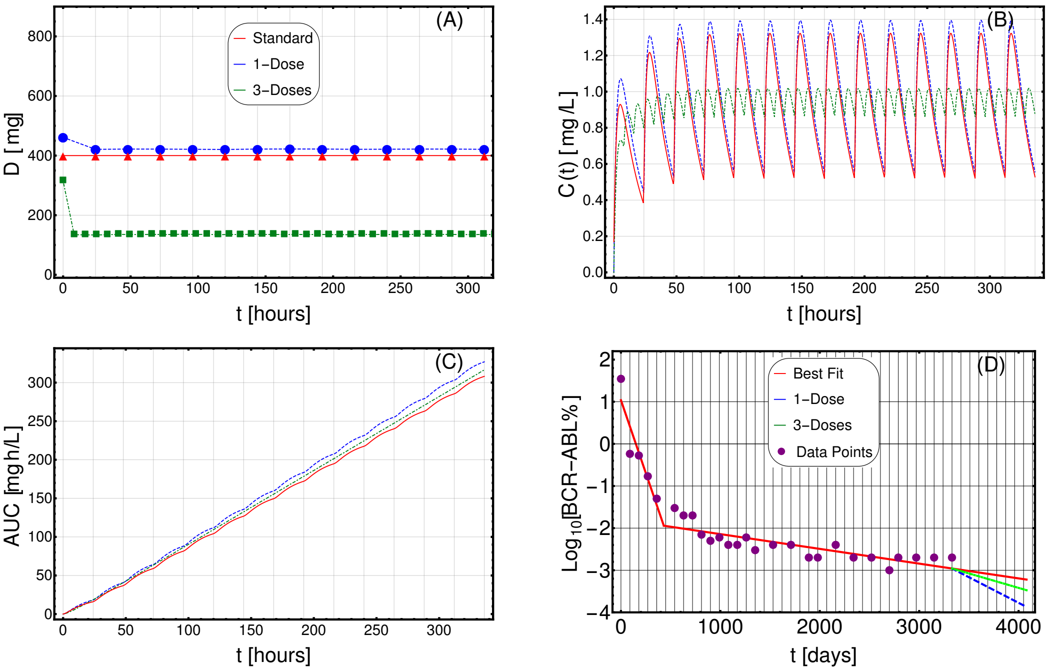

Figure S44: **Adjusting therapy for tumor burden minimization.** Imatinib administration optimized for tumor burden minimization in patient 0008 00005 BAT (male) from (Michor et al. (2005)), in the cases of: 1-dose/day (purple) and 3-doses/day (green), with $\phi = 60$, as compared to standard administration (red). In this case, the optimization is obtained on patient-specific PK and PD models. **(A)** Imatinib scheduled dosage in mg (y axis), displayed on 14 days (x axis). **(B)** Imatinib concentration in blood in $[mg/L]$ (y axis). **(C)** Temporal variation of the AUC in $[mg \cdot h/L]$ (y axis). **(D)** Longitudinal data points on tumor burden recorded are presented in purple. The best fit is shown with red lines. The slope of the right-most line is used to determine the cancer stem cell death rate and, in turn, the patient-specific PD parameters. The blue (green) line represents the predicted cancer subpopulation decay in case the 1-dose (3-doses) optimal administration

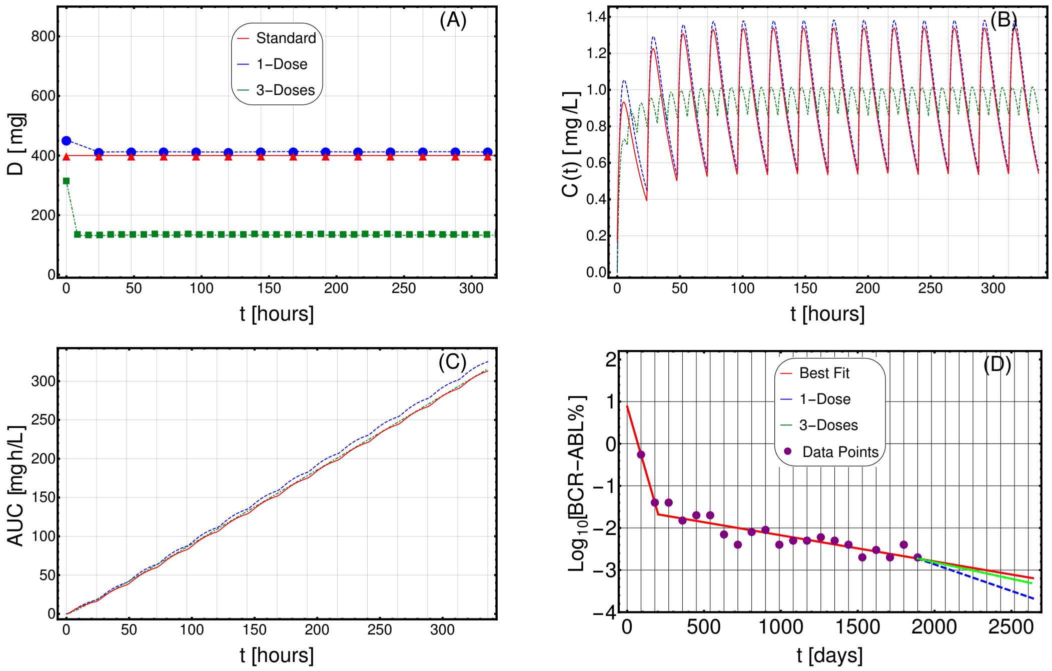

Figure S45: **Adjusting therapy for tumor burden minimization.** Imatinib administration optimized for tumor burden minimization in patient 0009 00003 LH (male) from (Michor et al. (2005)), in the cases of: 1-dose/day (purple) and 3-doses/day (green), with $\phi = 60$, as compared to standard administration (red). In this case, the optimization is obtained on patient-specific PK and PD models. **(A)** Imatinib scheduled dosage in mg (y axis), displayed on 14 days (x axis). **(B)** Imatinib concentration in blood in $[mg/L]$ (y axis). **(C)** Temporal variation of the AUC in $[mg \cdot h/L]$ (y axis). **(D)** Longitudinal data points on tumor burden recorded are presented in purple. The best fit is shown with red lines. The slope of the right-most line is used to determine the cancer stem cell death rate and, in turn, the patient-specific PD parameters. The blue (green) line represents the predicted cancer subpopulation decay in case the 1-dose (3-doses) optimal administration

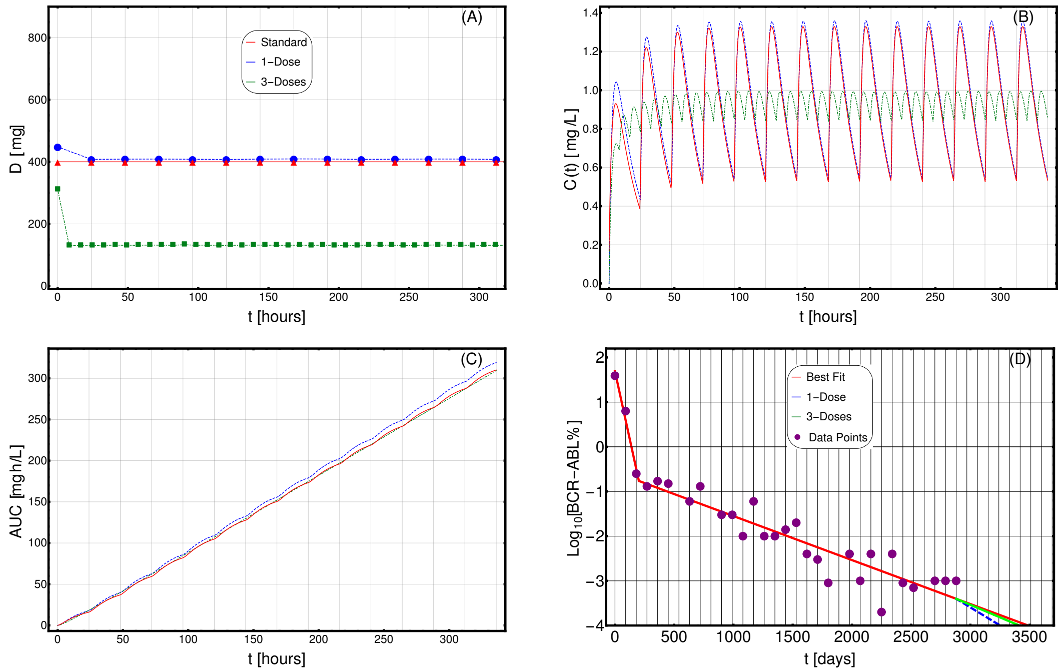

Figure S46: **Adjusting therapy for tumor burden minimization.** Imatinib administration optimized for tumor burden minimization in patient 0010 00001 HJ (male) from (Michor et al. (2005)), in the cases of: 1-dose/day (purple) and 3-doses/day (green), with $\phi = 60$, as compared to standard administration (red). In this case, the optimization is obtained on patient-specific PK and PD models. **(A)** Imatinib scheduled dosage in mg (y axis), displayed on 14 days (x axis). **(B)** Imatinib concentration in blood in $[mg/L]$ (y axis). **(C)** Temporal variation of the AUC in $[mg \cdot h/L]$ (y axis). **(D)** Longitudinal data points on tumor burden recorded are presented in purple. The best fit is shown with red lines. The slope of the right-most line is used to determine the cancer stem cell death rate and, in turn, the patient-specific PD parameters. The blue (green) line represents the predicted cancer subpopulation decay in case the 1-dose (3-doses) optimal administration

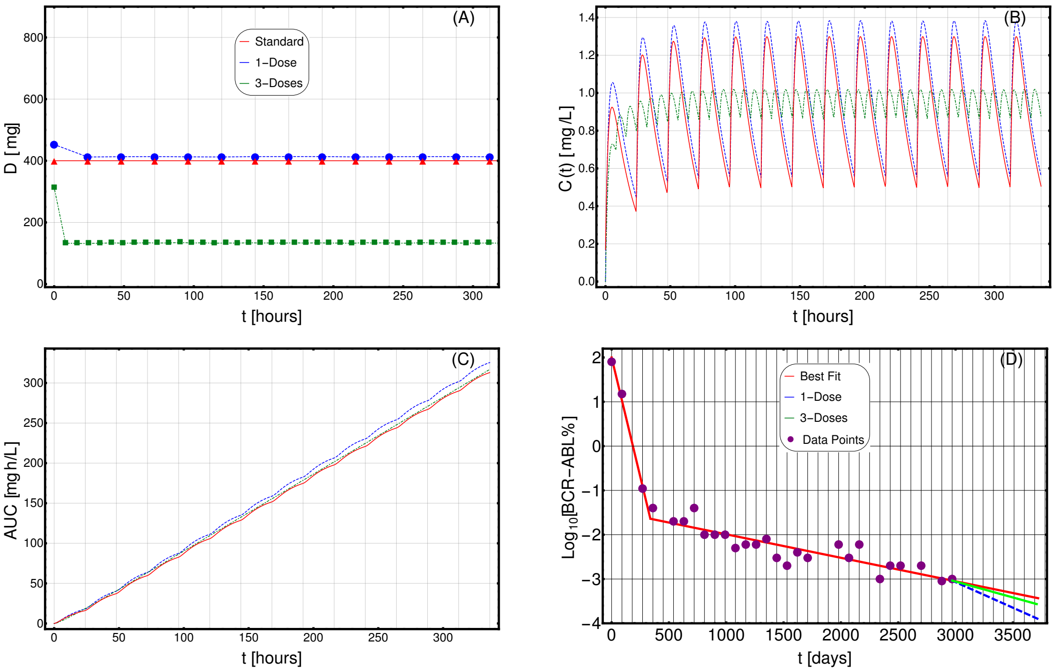

Figure S47: **Adjusting therapy for tumor burden minimization.** Imatinib administration optimized for tumor burden minimization in patient 0010 00002 GR (male) from (Michor et al. (2005)), in the cases of: 1-dose/day (purple) and 3-doses/day (green), with $\phi = 60$, as compared to standard administration (red). In this case, the optimization is obtained on patient-specific PK and PD models. **(A)** Imatinib scheduled dosage in mg (y axis), displayed on 14 days (x axis). **(B)** Imatinib concentration in blood in $[mg/L]$ (y axis). **(C)** Temporal variation of the AUC in $[mg \cdot h/L]$ (y axis). **(D)** Longitudinal data points on tumor burden recorded are presented in purple. The best fit is shown with red lines. The slope of the right-most line is used to determine the cancer stem cell death rate and, in turn, the patient-specific PD parameters. The blue (green) line represents the predicted cancer subpopulation decay in case the 1-dose (3-doses) optimal administration

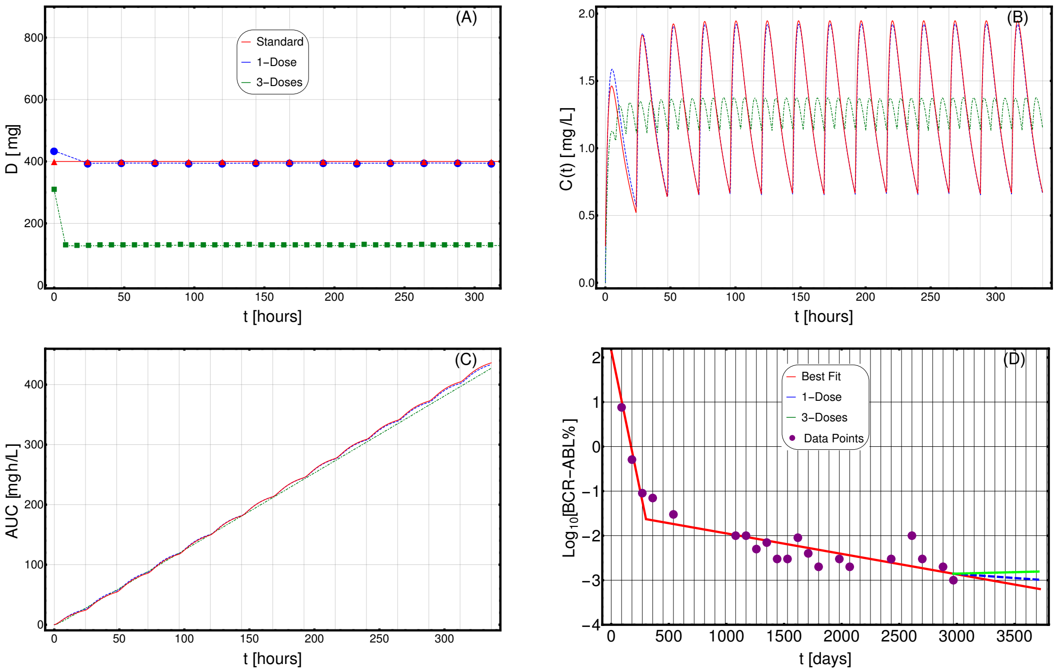

Figure S48: **Adjusting therapy for tumor burden minimization.** Imatinib administration optimized for tumor burden minimization in patient 0011 00001 CMC (female) from (Michor et al. (2005)), in the cases of: 1-dose/day (purple) and 3-doses/day (green), with $\phi = 75$, as compared to standard administration (red). In this case, the optimization is obtained on patient-specific PK and PD models. **(A)** Imatinib scheduled dosage in mg (y axis), displayed on 14 days (x axis). **(B)** Imatinib concentration in blood in $[mg/L]$ (y axis). **(C)** Temporal variation of the AUC in $[mg \cdot h/L]$ (y axis). **(D)** Longitudinal data points on tumor burden recorded are presented in purple. The best fit is shown with red lines. The slope of the right-most line is used to determine the cancer stem cell death rate and, in turn, the patient-specific PD parameters. The blue (green) line represents the predicted cancer subpopulation decay in case the 1-dose (3-doses) optimal administration

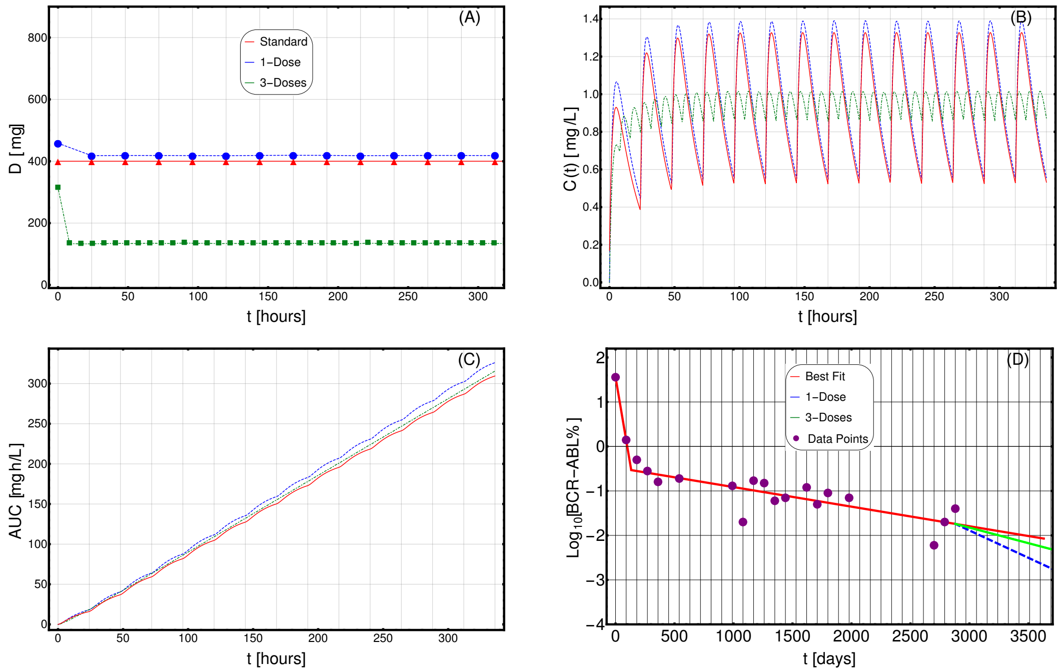

Figure S49: **Adjusting therapy for tumor burden minimization.** Imatinib administration optimized for tumor burden minimization in patient 0011 00005 SCW (male) from (Michor et al. (2005)), in the cases of: 1-dose/day (purple) and 3-doses/day (green), with $\phi = 60$, as compared to standard administration (red). In this case, the optimization is obtained on patient-specific PK and PD models. **(A)** Imatinib scheduled dosage in mg (y axis), displayed on 14 days (x axis). **(B)** Imatinib concentration in blood in $[mg/L]$ (y axis). **(C)** Temporal variation of the AUC in $[mg \cdot h/L]$ (y axis). **(D)** Longitudinal data points on tumor burden recorded are presented in purple. The best fit is shown with red lines. The slope of the right-most line is used to determine the cancer stem cell death rate and, in turn, the patient-specific PD parameters. The blue (green) line represents the predicted cancer subpopulation decay in case the 1-dose (3-doses) optimal administration

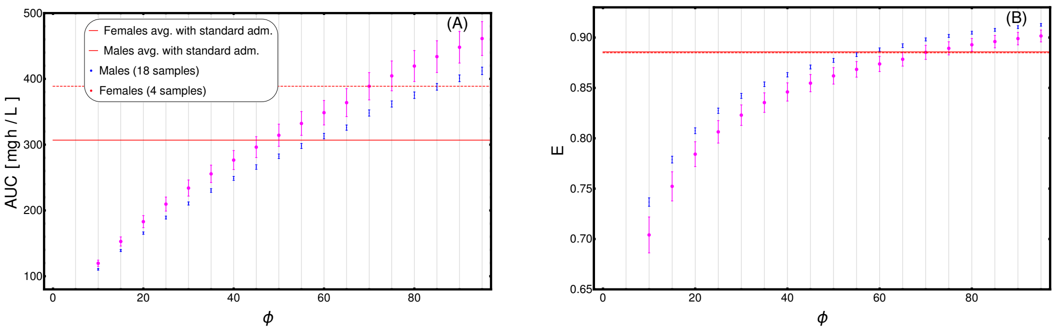

Figure S50: **Assessment of term weights in cost function definition (3-doses case).** The definition of the cost function for the adjusting treatment scenario requires to set the weights of the different terms. We here considered two terms, in order to: (i) minimize the tumor burden (weight W_1), and (ii) minimize the AUC (weight W_2) (see Materials and Methods for further details). We scanned the values of $\phi = \frac{W_1}{W_2}$ in the range $[10, 100]$, by repeatedly applying CT4TD to the 22-patients CML dataset from (Michor et al. (2005)). **(A)**. Distribution of the value of the AUC after 14-days of the optimized therapy retrieved by CT4TD (1-dose case), for distinct values of ϕ, with respect to the 22 samples in the datasets, divided in males (blue) and females (pink), and compared to the average AUC values returned by standard administration (400 mg Imatinib/day) in males (red solid line) and females (red dashed line). **(B)** Distribution of efficiency computed via Eq. (9) on the time-average concentration over 14 days of the optimized therapy retrieved by CT4TD (3-doses case), for distinct values of ϕ, and compared to the average efficiency in the standard administration scenario (solid and dashed red lines overlap).

3 TABLES

Table S1. Average parameters of PK model of Imatinib from (Widmer et al. (2006)).

Parameters	Value	Standard error	Units of measurement
k_a	0.61	30%	h^{-1}
CL	14.3	7.1%	Lh^{-1}
v	347	17.9%	L
$B\bar{W}$	70	Na	Kg
$A\bar{G}E$	50	Na	$Years$

Table S2. Summary of the demographic population PK parameters for Imatinib from (Widmer et al. (2006)).

Parameter	Value
k_a	0.437
θ_a	12.8
θ_b	258
θ_1	12.7
θ_2	0.8
θ_3	-2.1
θ_4	61.0

Table S3. Summary data analysis.

Code	$\alpha \quad [days]^{-1}$	$\beta \quad [days]^{-1}$	$T \quad [days]$	R^2	Index
0001 00002 RH	-0.00793675	-0.000430922	453	0.992329	A
0001 00004 AJR	-0.0335431	-0.000455828	112	0.99256	B
0001 00006 GMC	-0.0080923	-0.000454651	421	0.979851	C
0001 00009 MJG	-0.0153962	-0.000432947	218	0.985078	D
0002 00003 SMV	-0.000734153	-0.000223423	1710	0.992211	E
0002 00007 PMG	-0.0124442	-0.000680913	139	0.966844	F
0002 00008 SAT	-0.0204914	-0.000527755	121	0.987923	G
0003 00002 CL	-0.00158763	-0.000382959	1260	0.969166	H
0004 00003 CAR	-0.00249199	-0.00104476	540	0.980976	I
0004 00006 GM	-0.0186312	-0.00105491	105	0.987429	J
0006 00006 ML	-0.0154954	-0.00127265	90	0.949279	K
0006 00007 RJW	-0.01559	-0.000650637	224	0.994058	L
0007 00001 AJV	-0.0138184	-0.000720104	211	0.987969	M
0007 00002 DPS	-0.00937013	-0.000137684	408	0.918741	N
0008 00002 PWR	-0.0207251	-0.000485452	140	0.985233	O
0008 00003 LJG	-0.00583334	-0.000350359	204	0.991091	P
0008 00005 BAT	-0.00691467	-0.000350271	431	0.987516	Q
0009 00003 LH	-0.0126738	-0.000620824	202	0.994167	R
0010 00001 HJ	-0.0121572	-0.000984917	202	0.977477	S
0010 00002 GR	-0.0107683	-0.000530116	338	0.992	T
0011 00001 CMC	-0.0125545	-0.000459044	302	0.985232	U
0011 00005 SCW	-0.0157079	-0.000440187	133	0.948588	V

Table S4. PK personal parameters. Weights are taken from (McDowell et al. (2005))

Code	Sex	Age [Years]	Weight [Kg]	K_a	CL	V	EC_{50} $[mg/L]$
0001 00002 RH	M	65	88.6	0.437	17.3446	319	0.121311
0001 00004 AJR	M	55	88.8	0.437	17.8009	319	0.118284
0001 00006 GMC	M	35	86.6	0.437	18.2417	319	0.11573
0001 00009 MJG	M	53	88.8	0.437	17.8849	319	0.118019
0002 00003 SMV	M	24	80.7	0.437	17.6333	319	0.121672
0002 00007 PMG	M	64	88.6	0.437	17.3866	319	0.118461
0002 00008 SAT	M	39	86.6	0.437	18.0737	319	0.115963
0003 00002 CL	F	27	67.9	0.437	13.585	197	0.158738
0004 00003 CAR	F	23	67.9	0.437	13.753	197	0.148087
0004 00006 GM	M	49	89.1	0.437	18.1073	319	0.110521
0006 00006 ML	M	58	88.8	0.437	17.6749	319	0.110734
0006 00007 RJW	F	22	67.9	0.437	13.795	197	0.152898
0007 00001 AJV	M	50	88.8	0.437	18.0109	319	0.114397
0007 00002 DPS	M	22	80.7	0.437	17.7173	319	0.122035
0008 00002 PWR	M	55	88.8	0.437	17.8009	319	0.117983
0008 00003 LJG	M	55	88.8	0.437	17.8009	319	0.119356
0008 00005 BAT	M	59	88.8	0.437	17.6329	319	0.120372
0009 00003 LH	M	65	88.6	0.437	17.3446	319	0.119339
0010 00001 HJ	M	61	88.6	0.437	17.5126	319	0.114586
0010 00002 GR	M	63	88.6	0.437	17.3446	319	0.12028
0011 00001 CMC	F	52	75.9	0.437	12.535	197	0.169956
0011 00005 SCW	M	26	80.7	0.437	17.5493	319	0.119957

Table S5. Results of study of robustness. a and b refer to parameter of the curve $\Delta\mathcal{L}(\sigma_r)/\mathcal{L}_0 = a + b\sigma_r^2/r$ with $r = k_a, CL, v$. Plot of these curves are presented in fig. 2

Parameter	a	b	R^2
CL	-0.0051486	1.99948	0.997
v	-4.81123	212.498	0.997
k_a	-0.0179207	2.7182	0.998

Table S6. Configuration of RedCRAB

N. of SI	Basis	N. of basis in the 1th I.	N. of basis in the others I.s	N. of time points
50	Fourier	120	100	1001

REFERENCES

Castiglione, F. and Piccoli, B. (2007). Cancer immunotherapy, mathematical modeling and optimal control. *Journal of theoretical Biology* 247, 723–732

McDowell, A. M., Fryar, C., Hirsch, R., and Ogden, C. (2005). Anthropometric reference data for children and adults: U.S. population, 1999-2002. *Advance data* 361, 1–5

Michor, F., Hughes, T. P., Iwasa, Y., Branford, S., Shah, N. P., Sawyers, C. L., et al. (2005). Dynamics of chronic myeloid leukaemia. *Nature* 435, 1267

Widmer, N., Decosterd, L., Csajka, C., Leyvraz, S., Duchosal, M., Rosselet, A., et al. (2006). Population pharmacokinetics of imatinib and the role of $\alpha 1$-acid glycoprotein. *British journal of clinical pharmacology* 62, 97–112
